# Aminocarb Exposure Induces Cytotoxicity and Endoplasmic Reticulum Stress-Mediated Apoptosis in Mouse Sustentacular Sertoli Cells: Implications for Male Infertility and Environmental Health

**DOI:** 10.3390/biology13090721

**Published:** 2024-09-14

**Authors:** Sílvia Moreira, Ana D. Martins, Marco G. Alves, Luis Miguel Pastor, Vicente Seco-Rovira, Pedro F. Oliveira, Maria de Lourdes Pereira

**Affiliations:** 1 Department of Medical Sciences, University of Aveiro, 3810-193 Aveiro, Portugal; s.moreira@ua.pt (S.M.); marcoalves@ua.pt (M.G.A.); 2 CICECO-Institute of Materials, University of Aveiro, 3810-193 Aveiro, Portugal; 3 LAQV-REQUIMTE and Department of Chemistry, University of Aveiro, 3810-193 Aveiro, Portugal; cat.mar@ua.pt; 4 Departamento de Biología Celular e Histología, Faculdad de Medicina, IMIB-Arrixaca, Regional Campus of International Excellence “Campus Mare Nostrum”, Universidad de Murcia, 30120 Murcia, Spain; bioetica@um.es (L.M.P.); vicente.seco@gmail.com (V.S.-R.)

**Keywords:** male fertility, carbamates, sertoli cells, aminocarb, ER stress, apoptosis

## Abstract

**Simple Summary:**

Exposure to pesticides poses a significant threat to male fertility by compromising crucial cells involved in spermatogenesis. Although aminocarb is a widely used carbamate insecticide, little is known about its impact on male fertility and, consequently, on sustentacular Sertoli cells. In this study, we investigated the effects of increasing concentrations of aminocarb on a mouse Sertoli cell line, TM4. Assessments included analyses of cellular proliferation and viability, membrane integrity, mitochondrial biogenesis and membrane potential and expression and the activity of apoptotic proteins, as well as oxidative stress. Our findings revealed a dose-dependent reduction in the proliferation and viability of TM4 cells following exposure to aminocarb. Moreover, exposure to 5 μM of aminocarb induced depolarization of mitochondria membrane potential, and a decrease in the ratio of phosphorylated eIF2α to total eIF2α, suggesting heightened endoplasmic reticulum stress via the activation of the eIF2α pathway. An elevation in both caspase-3 protein levels and activity was also observed for the cells under this same aminocarb concentration, thus indicating an apoptotic induction. Altogether, our results demonstrate that aminocarb serves as an apoptotic inducer for mouse sustentacular Sertoli cells in vitro, suggesting its potential to modulate independent pathways of the apoptotic cascade.

**Abstract:**

Exposure to pesticides, poses a significant threat to male fertility by compromising crucial cells involved in spermatogenesis. Aminocarb, is a widely used carbamate insecticide, although its detrimental effects on the male reproductive system, especially on sustentacular Sertoli cells, pivotal for spermatogenesis, remains poorly understood. In this study, we investigated the effects of escalating concentrations of aminocarb on a mouse Sertoli cell line, TM4. Assessments included cytotoxic analysis, mitochondrial biogenesis and membrane potential, expression of apoptotic proteins, caspase-3 activity, and oxidative stress evaluation. Our findings revealed a dose-dependent reduction in the proliferation and viability of TM4 cells following exposure to increasing concentrations of aminocarb. Notably, exposure to 5 μM of aminocarb induced depolarization of mitochondria membrane potential, and a significant decrease in the ratio of phosphorylated eIF2α to total eIF2α, suggesting heightened endoplasmic reticulum stress via the activation of the eIF2α pathway. Moreover, the same aminocarb concentration was demonstrated to increase both caspase-3 protein levels and activity, indicating an apoptotic induction. Collectively, our results demonstrate that aminocarb serves as an apoptotic inducer for mouse sustentacular Sertoli cells in vitro, suggesting its potential to modulate independent pathways of the apoptotic cascade. These findings underscore the deleterious impact of aminocarb on spermatogenic performance and male fertility, highlighting the urgent need for further investigation into its mechanisms of action and mitigation strategies to safeguard male fertility.

## 1. Introduction

Infertility, defined as the inability to conceive after at least 12 months of regular and unprotected sexual intercourse [1], is a major health problem worldwide and is estimated to affect 8–12% of couples in reproductive age. Specifically, infertility lies solely with the man in 20–30% of cases, and a male cause is contributory in a further 20% [2]. Several causes and risk factors can contribute to the increasing incidence of male infertility, among which is environmental or occupational exposure to toxic chemicals [3]. Indeed, various studies have already demonstrated the capacity of these compounds to induce damages on the male reproductive system in several ways, ranging from direct impact on testicular cells [4], leading them to apoptosis, to altered testicular tissue morphology [5] or spermatogenesis [6].

Sustentacular Sertoli cells are one of the major testicular cell types [3], often being referred to as “nurse cells” due to the fact that fully differentiated sustentacular Sertoli cells are responsible for providing support to developing germ cells. In fact, sustentacular Sertoli cells are crucial for the spermatogenic process, as they not only produce lactate and other metabolic intermediates, such as amino acids, carbohydrates, lipids, vitamins and metal ions used by the developing germ cells to produce ATP, but also create the blood–testis barrier, which is essential for the proper development of germ cells into fully functional sperm [7]. Interestingly, a condition in which the testis contained germ cells but not sustentacular Sertoli cells was never observed, thus corroborating the importance of these cells to male fertility [7,8,9]. However, sustentacular Sertoli cells are targeted by external and internal adverse conditions, such as hormonal deregulation, diseases, oxidative stress and environmental factors, among others, which can impair their function, and thereby compromise the appropriate environment necessary for the proper development of germ cells [7].

Regarding specifically the impact of pesticides on sustentacular Sertoli cells, it has been demonstrated that these xenobiotics can promote cell apoptosis, which can be activated by three pathways, including the mitochondrial pathway, death receptor pathway and endoplasmic reticulum (ER) pathway [10]. In the mitochondrial pathway, also known as the intrinsic pathway, as the name indicates, mitochondria is the central organelle governed by pro- and anti-apoptotic B-cell lymphoma-2 (Bcl-2) family members, and the apoptotic process is executed by a family of cysteine proteases, known as caspases. Briefly, when apoptosis is induced, mitochondrial membranes suffer changes, like permeabilization of the outer mitochondrial membrane and loss of inner mitochondrial membrane potential (∆ψm), which becomes depolarized. Subsequently, cytochrome c is released into the cytoplasm, which leads to a cascade that activates executioner caspase-3, leading to apoptosis [11]. On the other hand, the death receptor pathway, also known as the extrinsic apoptotic pathway [12,13], is initiated by the binding of an extracellular death ligand to its cell surface death receptors (like Fas/CD95, DR4 and DR5), thus influencing the intracellular apoptotic adaptor FADD protein, which proceeds through caspase-8 by mediating cleavage and activation of effector caspases [14]. Furthermore, under the stimulation of exogenous chemicals like pesticides, protein folding in the ER may be arrested, culminating in the accumulation of misfolded proteins inside the lumen of the ER, thus causing ER stress, which can also lead to apoptosis [15].

Carbamate pesticides, developed in the 1950s, are one of the classes of pesticides most used in agriculture, floriculture and forestry, worldwide, due to their lack of residue persistency in the environment. Notwithstanding, these compounds lack species selectivity, and pose a serious threat to the environment and to the health of humans and other animals, since they are extremely toxic [16]. As such, the use of these chemicals should be taken seriously, in accordance with the One Health concept, which is defined as an approach to address health threats at the human–animal–environment interface based on collaboration, communication and coordination across all relevant sectors and disciplines, with the ultimate goal of achieving optimal health outcomes for both people and animals, being applicable at the subnational, national, regional and global level [17]. Aminocarb, also known as 4-dimethylamino-3-methylphenyl N-methylcarbamate, is a carbamate insecticide particularly used to control the spruce budworm (*Choristoneura fumiferana*) in eastern Canada [18]. Despite its popularity, this compound is highly toxic to mammals and some types of fish [19], and very few studies have reported on its impact on the male reproductive system. As such, due to the crucial roles of sustentacular Sertoli cells in male fertility, and since they are targets of external adverse conditions like environmental factors [7], we decided to focus our attention in this study on the impact of aminocarb on these cells. For that, the TM4 mouse Sertoli cell line was used, which has been developed as a suitable model for in vitro research mostly due to the similarities to primary sustentacular Sertoli cells [20]. These cells were exposed to increasing concentrations of aminocarb and the cytotoxic effects induced by aminocarb on the TM4 cell line were investigated, with a particular focus on the eliciting of selected apoptotic pathways.

## 2. Materials and Methods

### 2.1. Chemicals

Aminocarb (45322-50MG) was purchased from Sigma–Aldrich (St. Louis, MO, USA) in powder form (50 mg) and dissolved in ethanol. Fetal Bovine Serum (FBS), Dulbecco’s Modified Eagle Medium Ham’s Nutrient Mixture F12 (DMEM:Ham’s F12) and trypsin were all purchased from Sigma–Aldrich (St. Louis, MO, USA). 3-(4,5-dimethylthiazol-2-yl)-2,5-diphenyltetrazolium bromide (MTT) was purchased from Amresco (Solon, OH, USA). Sulforhodamine B (SRB) was purchased from Biotium (Hayward, CA, USA). An LDH-Cytox^TM^ Assay kit was acquired from BioLegend (San Diego, CA, USA). A NZY Tissue gDNA Isolation kit (MB13502), a NZY Total RNA Isolation kit (MB13402) and a NZY M-MuLV Reverse Transcriptase were purchased from NZY tech (Lisboa, Portugal). A PowerTrack SYBER Green Master Mix (A46111) was purchased from Thermo Fisher Scientific. A NZY DNA ladder IV (MB05801) was purchased from NZY tech (Lisboa, Portugal). A Pierce^TM^ BCA^®^ Protein Assay Kit was obtained from Thermo Scientific (Waltham, MA, USA). Dried milk was obtained from Regilait (SaintMartin–Belle–Roche, France). A Clarity Western ECL substrate was purchased from Bio–Rad (Hercules, CA, USA). All other chemicals were obtained from Merck–Sigma–Aldrich (Darmstadt, Germany), otherwise stated.

### 2.2. Experimental Design

The three cytotoxic assays, as well as the following experiments, were performed using immortalized sustentacular Sertoli cells from a mouse (TM4 cell line) (CRL-1715™), previously purchased from ATCC (LGC Standards, Middlesex, UK). The cells were cultured and handled according to the ATCC protocol and original guidelines [21]. The cells were allowed to grow until they were 100% confluent in DMEM:Ham’s F12 medium (1:1), supplemented with 15 mM HEPES, 100 U/mL penicillin, 100 µg/mL streptomycin sulfate, 2.5 µg/mL fungizone, 50 mg/mL gentamicin and 10% FBS, pH 7.4, under standard conditions (37 °C, 5% CO_2_). Subsequently, the cells were cultured for 24 h with increasing concentrations of aminocarb (0.05, 0.5, 5, 50 and 500 µM), and compared to a control group (solubilization vehicle only). Considering the general scarcity of studies on the impact of aminocarb on the male reproductive system, the doses used in this study were selected based on literature reporting the blood concentrations of other chemicals belonging to the carbamate family [22] and on a previous study that reported different aminocarb concentrations in water and around sprayed areas [23]. After treatment, cells were collected and harvested for protein extraction and quantification.

### 2.3. Evaluation of Cell Proliferation through the SRB Assay

In order to assess TM4 cell proliferation we resorted to the SRB assay. This dye binds stoichiometrically to basic amino acid residues in proteins, by the sulfonyl groups, thus directly representing the total protein mass, and so it is proportional to the cell number [24]. Cells were seeded in a 24 well culture plate, left to grow to 70% confluence, and treated with the different concentrations of aminocarb or vehicle over a 24 h period at 37 °C, 5% CO_2_. After treatment, cells were washed with phosphate buffered saline (PBS) and fixed in 1% acetic acid in methanol at −20 °C for 1 h. Then, cells were stained for 1 h with 0.05% (*w*/*v*) SRB dissolved in 1% acetic acid at 37 °C. Unbound SRB was removed by washing the cells with a 1% acetic acid solution. Finally, 250 μL of 10 mM Tris (pH = 10) was added to dissociate the cell-bound dye and, after 10 min of shaking, 100 μL of each well was transferred to a 96 well plate. The optical densities of the resulting media were determined at 490 nm. To obtain the concentration response, we defined the cell growth of the control group as 1 and calculated the cell growth of treated groups after subtracting the optical densities, due to cell density before the treatment to each experimental condition and dividing by the value of the control group, as described previously [24,25]. The results are presented as a variation to control, with values > 1 representing an increase in cell proliferation and values < 1 representing a decrease in cell proliferation, with negative values representing increased cell death.

### 2.4. Evaluation of Mitochondrial Function through the MTT Assay

In order to evaluate the TM4 cells’ viability, we chose to perform the MTT assay. The MTT compound is reduced by viable cells that possess NAD(P)H-dependent oxidoreductase enzymes to formazan precipitate, which is directly proportional to the number of viable cells [24]. Cells were seeded in a 48 well culture plate, left to grow to 70% confluence, and then exposed to the different concentrations of aminocarb or vehicle for 24 h at 37 °C, 5% CO_2_. Thereafter, cells were washed with PBS and 150 μL of medium (FBS free) plus 15 μL of MTT (5 mg/mL) dissolved in PBS were added to each well. Cells were then protected from light and incubated for 2 h at 37 °C. Posteriorly, cells were washed with DMSO and 100 μL from each well were transferred to a 96 well culture plate. A blank was made with DMSO, and the absorbance was read at 570 nm. Cell viability was expressed in fold variation to control.

### 2.5. Evaluation of Membrane Damages through the Lactate Dehydrogenase (LDH) Assay

In order to evaluate possible damages on cellular membranes induced by exposure to aminocarb, the LDH-Cytox^TM^ Assay kit was used, which allowed us to measure extracellular LDH as a known biomarker for cytotoxicity. Cells were seeded in a 48 well culture plate, left to grow to 70% confluence, and then treated with the different concentrations of aminocarb or vehicle for 24 h at 37 °C, 5% CO_2_. Then, 100 μL of each sample medium was transferred to a 96 well plate and 100 μL of reaction mixture was added. Next, the plate was incubated at 37 °C for 30 min, protected from light. The reaction was stopped by adding 50 μL of stop solution and the optical densities of the resulting medium were determined at 490 nm. Cytolysis was expressed in fold variation to control.

### 2.6. DNA and RNA Extraction

Total DNA and RNA extractions from TM4 cells were performed using a NZY Tissue gDNA Isolation Kit and a NZY Total RNA Isolation Kit, respectively, according to the manufacturer’s instructions. The extracted DNA and RNA were quantified using a NanoDrop One Spectrophotometer (Thermo Fisher Scientific, Waltham, MA, USA).

### 2.7. Polymerase Chain Reaction

cDNA was obtained from extracted total RNA using NZY M-MuLV Reverse Transcriptase. Specific cDNA fragments were amplified using a designed exon–exon spanning primer set (Table 1), with optimal annealing temperatures. Sirtuin 1 (*Sirt1*), peroxisome proliferator-activated receptor γ coactivator 1-α (*PGC-1α*), nuclear respiratory factor 1 (*NRF1*), transcription factor A, and mitochondrial (*TFAM*) mRNA levels were evaluated in mSCs, and β-2-microglobulin (*β2M*) transcript levels were used to normalize expression. Target genes, sequences and annealing temperatures are presented in Table 1. The efficiency of the amplification and quantitative PCR (qPCR) experiments were carried out with a CFX Duet Real-time PCR system (Biorad, Hercules, CA, USA). The fold variation of the expression of target genes was calculated using the mathematical model suggested by Pfaffl [26] in the formula: 2^−∆Ct^, where ∆Ct is the deviation of the control-sample of the reference of the transcript gene.

### 2.8. Determination of Mitochondrial DNA Copy Number (mtDNAcn)

In order to determine mtDNAcn, we resorted to qPCR analysis in a CFX Duet Real-time PCR system (Biorad, Hercules, CA, USA), as described [27]. Annealing temperatures and primer sequence are presented in Table 1. To quantify the relative mtDNAcn, we used the model proposed by Pfaffl (2^−∆Ct^), where Ct value differences between NADH-ubiquinone oxidoreductase chain 1 (*ND1*) and nuclear encoded β-2-microglobulin (*β2M_nc_)* were used.

### 2.9. JC-1 Assay for ∆ψm

The ∆ψm of TM4 cells was evaluated by using the lipophilic probe 5-5′,6-6′-tetrachloro-1,1′,3,3′- tetraethylbenzimidazolcarbocyanine iodide (JC-1) (T3168, Invitrogen™, Carlsbad, CA, USA). Briefly, cells were seeded in a 96 well culture plate, left to grow to 80% confluence, and then treated with the different concentrations of aminocarb (0.5 and 5 μM) or vehicle for 24 h at 37 °C, 5% CO_2_. After treatment, the cells were washed with PBS and incubated with JC-1 (2 µg/mL) for 30 min at 37 °C. After the incubation period, cells were washed with PBS two times, and 100 μL of culture medium were added to each well. The fluorescence of the JC-1 monomers (ex 485/530 nm; excitation/emission) and JC-1 aggregates (535/590 nm; excitation/emission) were assessed by TECAN Infinite 200 PRO microplate reader (Männedorf, Switzerland). The ratio between JC-1 J-aggregates/monomers was calculated and used as a ∆ψm marker, where an increased/decreased ratio was considered indicative of hyperpolarization/depolarization, respectively [28].

### 2.10. Protein Extraction and Quantification

The protein total was extracted from TM4 cells using Triton-X buffer (1% Triton-X 100, HEPES 10 mM, EGTA 0.5 mM, pH = 7.3) that was supplemented with 100 mM PMSF, 1% protease inhibitor cocktail, and 100 mM sodium orthovanadate. A total of 50 μL of this lysis buffer was added to the cell homogenate, and the resulting solution was agitated for 10 min at room temperature, followed by 20 min of centrifugation at 14,000× *g* at 4 °C. Total protein quantification was performed using the PierceTM Microplate BCA Protein Assay kit according to manufacturer instructions. The calibration curve was calculated by using increasing concentrations of BSA as standards. Sample absorbances were measured at 560 nm using a multiplate reader (MultiSkan Go, Thermo Fisher Scientific, Vantaa, Finland).

### 2.11. Western Blotting

Protein samples (50 μg) were mixed with sample buffer (1.5 M Tris.HCl pH = 6.8, 10% glycerol, 2% SDS, 5% 0.01% β-mercaptoethanol and bromophenol blue) and denatured for 10 min at 37 °C. Proteins were fractioned on a SDS-PAGE system with TGX^TM^ Stain-Free Acrylamide gel (Bio–Rad), 90 V gel for 90 min, at room temperature and transferred to polyvinylidene difluoride membranes in a Trans-Blot^®^ Turbo^TM^ transfer System (Bio–Rad; 1.3 A, up to 25 V, 10 min). The protein total was assessed by fluorescence using the TGX^TM^ Stain-Free protocol and was used as a loading control, as described previously [29], for it was shown to be superior against housekeeping proteins [30]. The membranes were blocked for 90 min in a 5% non-fat milk solution at room temperature. Membranes were incubated overnight at 4 °C with primary antibodies listed in Table 2. Immune-reactive proteins were detected with goat anti-rabbit. Membranes were reacted with a Clarity Western ECL substrate and read with the Bio–Rad ChemiDoc version 2.4.0.03 (Bio–Rad, Hemel Hempstead, UK). Densities of each band were obtained using the Image Lab^TM^ Software version 6.0.0 (Bio–Rad, Hemel Hempstead, UK) by standard methods. The band density obtained was normalized with the value of total proteins and expressed as a fold variation to the control group.

### 2.12. Caspase-3 Activity Assay

Caspase-3 activity was assessed spectrophotometrically by determining the cleavage of the respective colorimetric substrate. In brief, 25 µg of proteins were incubated in assay buffer (25 mM HEPES, pH 7.5, 0.1% CHAPS, 10% sucrose and 10 mM DTT) with 100 µM of caspase-3 substrate (Ac-DEVD-pNA) for 2 h at 37ºC. The caspase-3 activity was determined by the detection of p-nitroanilide chromophore, measured at 405 nm in a spectrophotometer. The method was calibrated with known concentrations of p-nitroanilide. The activity was calculated as units per milligram of protein using the molar extinction factor (ε) of pNA and expressed as a fold variation to the control group.

### 2.13. Oxidative Stress Evaluation

Levels of lipid peroxidation, protein carbonylation and protein nitration were evaluated by immunoblotting. For lipid peroxidation and protein nitration levels detection, 10 µg of protein was diluted in PBS to a final volume of 100 µL and transferred to nitrocellulose membranes through a slot–blot system. For protein carbonylation evaluation, we used the protocol described in [31]. In brief, 5 µg of protein was diluted in PBS to a final volume of 20 µL. To each sample was added 20 µL of 12% SDS solution for protein denaturation and carbonyl groups exposure. Subsequently, 40 µL of 20 mM 2,4-dinitrophenylhydrazine (DNPH) in 10% trifluoroacetic acid (TFA) was added, and the reaction was allowed to occur for 30 min at room temperature, in the dark. To stop the reaction between carbonyl groups and DNPH, 30 µL of 2M Tris with 18% β-mercaptoethanol solution was added. 2.4 µL of the derivatized samples were diluted in 107.6 µL of PBS and transferred to PVDF membranes through a slot–blot system. Membranes were then blocked with a blocking solution [5% non-fat milk in NaCl, Tris-Buffered Saline and 0.1% Tween 20 (TBS-T)] for 1 h and incubated overnight with the correspondent primary antibody. For lipid peroxidation evaluation, the primary antibody used was Anti-4-Hydroxynonenal (4- HNE) (1:1000 diluted in 1% BSA TBS-T; AB5605, Sigma-Aldrich, St. Louis, MO, USA). For protein nitration evaluation, the primary antibody used was Anti-Nitro-Tyrosine (1:1000 diluted in 1% BSA TBS-T; 9691S, Cell Signaling Technology, Danvers, MA, USA). For protein carbonylation evaluation, the primary antibody used was anti-dinitrophenol (DNP) (1:5000 diluted in 1% BSA TBS-T; D9656-2ML, Sigma-Aldrich, St. Louis, MO, USA). Membranes were washed with TBS-T and incubated with an appropriate secondary antibody for 1 h, Anti-Goat 1:1000 diluted in 1% non-fat milk TBS-T (A4187, Sigma-Aldrich, St. Louis, MO, USA), and Anti-Rabbit 1:1000 diluted in 1% non-fat milk TBS-T (AB6721, Abcam, Cambridge, UK). Immuno-reactive proteins were detected through reaction with an ECL substrate (BIO-RAD, Hercules, CA, USA), and read with the Bio–Rad ChemiDoc version 2.4.0.03 (Bio–Rad, Hemel Hempstead, UK). Densities of each band were obtained using the Image Lab^TM^ Software version 6.0.0 (Bio–Rad, Hemel Hempstead, UK) by standard methods. The band density obtained was expressed as a fold variation to the control.

### 2.14. Statistical Analysis

Data are shown as the mean ± SEM or the median ± max/min values (represented by box plots) (n = 6 for each condition, n refers to distinct passages of cell culture). In the box plots presented in these experiments, the whiskers represent the minimum and maximum, while the box represents the lower and upper quartiles, with the median represented by the middle line. A one-way ANOVA followed by Fisher’s post hoc test was used to assess variation between experimental groups. A value of *p* < 0.05 was considered significantly different. Statistical analyses were performed using GraphPad Prism 8 (GraphPad Software, San Diego, CA, USA).

## 3. Results

### 3.1. Exposure to Aminocarb Decreases the Proliferation of TM4 Cells

**Figure 1:**
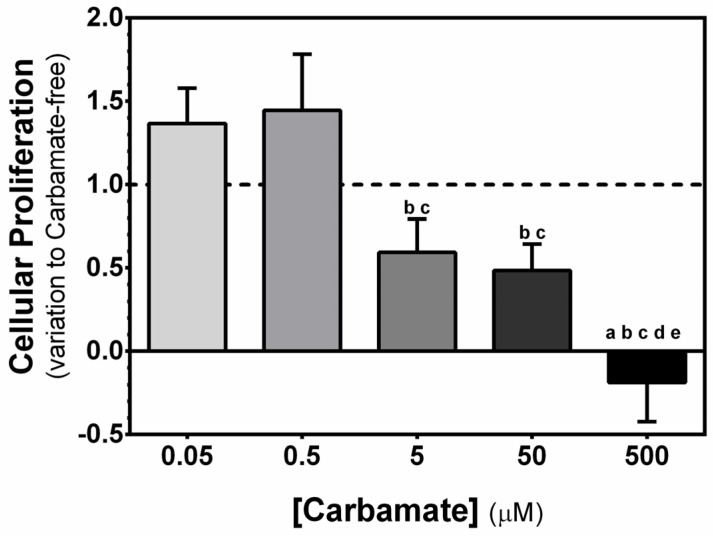
Effect of increasing concentrations of aminocarb on TM4 cells’ proliferation. The figure shows pooled data of independent experiments, indicating proliferation of TM4 cells cultured for 24 h in the absence or in the presence of aminocarb (0.05, 0.5, 5, 50 and 500 µM). Results were calculated as variation to control and presented as mean ± SEM (n = 6 for each condition). Duplicates were performed for each individual experiment. The dashed line represents the control group. The statistical significance was assessed by a one-way ANOVA followed by Fisher’s LSD test. Significantly different results (*p* < 0.05) are indicated as: a—relative to control group, b—relative to 0.05 µM group, c—relative to 0.5 µM group, d—relative to 5 µM group, e—relative to 50 µM group.

To evaluate the cytotoxic effects of exposure to increasing concentrations of aminocarb on TM4 cells, three different cytotoxicity assays were used. First, we evaluated the cellular proliferation by means of the SRB assay [24,25]. Our results showed that exposure to aminocarb 5 and 50 µM decreased TM4 cells’ proliferation (0.59 ± 0.20 and 0.48 ± 0.16—variation to control, *p* = 0.0144, *p* = 0.0079, *p* = 0.0063, and *p* = 0.0034, respectively) when compared to cells exposed to lower concentrations, 0.05 and 0.5 µM (1.37 ± 0.21 and 1.44 ± 0.34—variation to control, respectively). Additionally, exposure to aminocarb 500 µM also decreased TM4 cell proliferation (-0.19 ± 0.24—variation to control, *p* = 0.002) when compared to both the control group cells (1.00 ± 0.00—variation to control) and to cells exposed to lower concentrations, 0.05 µM (*p* < 0.0001), 0.5 µM (*p* < 0.0001), 5 µM (*p* = 0.0081) and 50 µM (*p* = 0.0197) (Figure 1).

### 3.2. Exposure to Aminocarb Decreases the Viability of TM4 Cells

**Figure 2:**
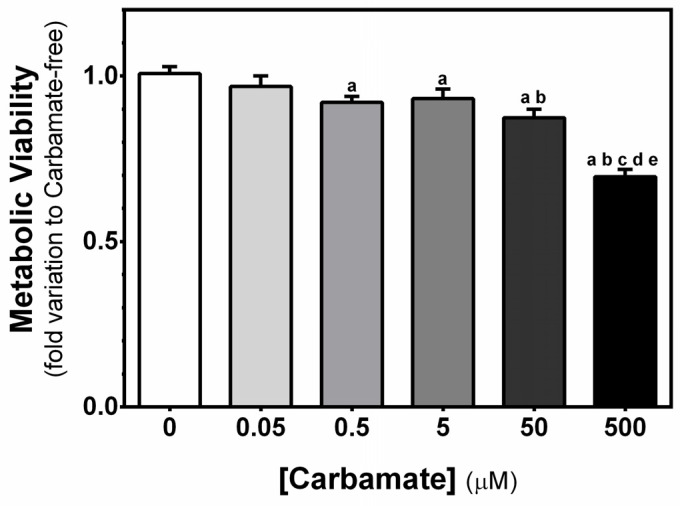
Effect of increasing concentrations of aminocarb on TM4 cells’ viability. The figure shows pooled data of independent experiments, indicating TM4 cell viability cultured for 24 h in the absence or in the presence of aminocarb (0.05, 0.5, 5, 50 and 500 µM). Results were calculated as fold variation to control and presented as mean ± SEM (n = 6 for each condition). Duplicates were performed for each individual experiment. The statistical significance was assessed by a one-way ANOVA followed by Fisher’s LSD test. Significantly different results (*p* < 0.05) are indicated as: a—relative to control group, b—relative to 0.05 µM group, c—relative to 0.5 µM group, d—relative to 5 µM group, e—relative to 50 µM group.

To better understand the cytotoxic effects of aminocarb exposure on TM4 cells, we assessed metabolic viability by means of the MTT assay [24]. Similarly to the previous results, exposure to increasing concentrations of aminocarb resulted in a reduction of the metabolic viability of TM4 cells for 0.5, 5, 50 and 500 µM (0.92 ± 0.02, 0.93 ± 0.03, 0.87 ± 0.03 and 0.70 ± 0.02—fold variation to control, *p* = 0.0207, *p* = 0.0422, *p* = 0.0007, and *p* < 0.0001, respectively) when compared to the control group cells (1.01 ± 0.02—fold variation to control). Exposure to aminocarb 50 µM also demonstrated a reduction to the metabolic viability of TM4 cells when compared to cells exposed to 0.05 µM (0.97 ± 0.03—fold variation to control, *p* = 0.0120). Moreover, cells exposed to aminocarb 500 µM also presented reduced metabolic activity, when compared to 0.05 µM (*p* < 0.0001), 0.5 µM (*p* < 0.0001), 5 µM (*p* < 0.0001) and 50 µM (*p* < 0.0001) (Figure 2).

### 3.3. Exposure to Aminocarb Presents a Tendency to Increase Membrane Damages in a Dose-Dependent Manner

**Figure 3:**
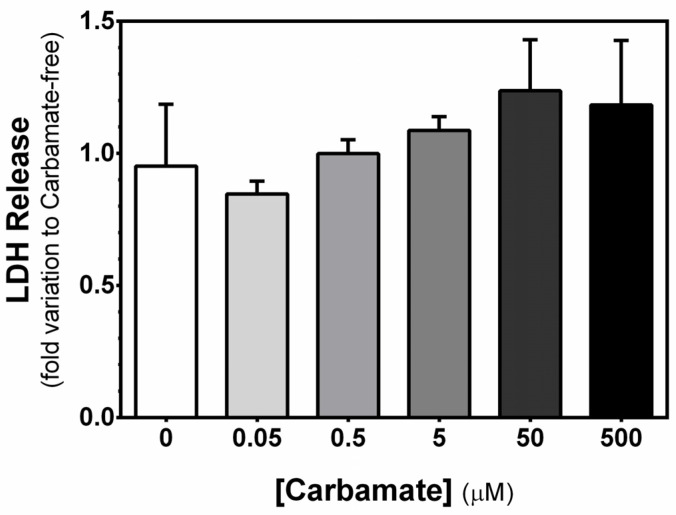
Effect of increasing concentrations of aminocarb on TM4 cells’ LDH release. The figure shows pooled data of independent experiments, indicating LDH release by TM4 cells cultured for 24 h in the absence or in the presence of aminocarb (0.05, 0.5, 5, 50 and 500 µM). Results were calculated as fold variation to control and presented as mean ± SEM (n = 6 for each condition). Duplicates were performed for each individual experiment. The statistical significance was assessed by a one-way ANOVA followed by Fisher’s LSD test.

Finally, we assessed the impact of exposure to increasing concentrations of aminocarb on TM4 cell cytolysis, through the measurement of the release of the cytoplasmic enzyme LDH. However, we did not observe any significant alteration to the LDH release rate into the extracellular medium after the exposure of TM4 cells to 0.05 μM aminocarb (0.85 ± 0.05—fold variation to control, *p* = 0.6500), 0.5 μM aminocarb (1.00 ± 0.05—fold variation to control, *p* = 0.8381), 5 μM aminocarb (1.09 ± 0.05—fold variation to control, *p* = 0.5614), 50 μM aminocarb (1.24 ± 0.19—fold variation to control, *p* = 0.2252), or 500 μM aminocarb (1.18 ± 0.24—fold variation to control, *p* = 0.3226), when compared to the control group cells (0.95 ± 0.23—fold variation to control) (Figure 3).

### 3.4. Exposure to Aminocarb Does Not Affect Mitochondrial Biogenesis But Alters Mitochondrial Membrane Potential (∆ψm)

**Figure 4:**
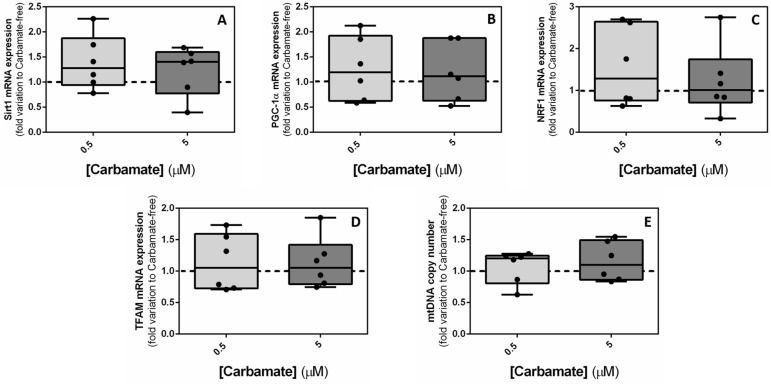
Effect of 0.5 μM and 5 μM of aminocarb in mitochondria biogenesis-related genes (**A**–**D**) and mtDNAcn (**E**), after 24 h of treatment. The figure shows pooled data of independent experiments, indicating mRNA transcript levels of Sirtuin 1 (**A**), peroxisome proliferator-activated γ receptor coactivator 1-α (PGC-1α) (**B**), nuclear respiratory factor 1 (NRF1) (**C**), and mitochondrial transcription factor A (TFAM) (**D**), and mtDNA copies (mtDNA) (**E**) in TM4 cells detected by quantitative PCR. Results were calculated as fold variation to control and are expressed as median ± max/min values (n = 6 for each condition). The dashed line represents the control group. The statistical significance was assessed by a one-way ANOVA followed by Fisher’s LSD test.

Based on our first results from the cytotoxicity assays, we decided to continue our study with only two aminocarb concentrations. For this purpose, the two intermediate ones were chosen (0.5 and 5 µM), since the exposure of TM4 cells to the two highest concentrations (50 and 500 μM) greatly reduced the number of cells. Next, due to the observed impact of aminocarb on mitochondrial dehydrogenase activity, as assessed by the MTT assay, we decided to further explore the consequences of exposure to aminocarb on mitochondria, and analyzed genes related with mitochondrial biogenesis, such as *Sirt1*, *PGC-1α*, *NRF1* and *TFAM*. Additionally, we quantified mitochondrial DNA content (*ND1* gene). Notwithstanding, our results did not show any significant differences in the expression of *Sirt1* when TM4 cells were exposed to 0.5 μM aminocarb (1.39 ± 0.22—fold variation to control, *p* = 0.1661) or 5 μM aminocarb (1.23 ± 0.20—fold variation to control, *p* = 0.4106), compared to the control group cells (1.00 ± 0.14—fold variation to control) (Figure 4—panel A); *PGC-1α* when TM4 cells were exposed to 0.5 μM aminocarb (1.27 ± 0.26—fold variation to control, *p* = 0.4200) or 5 μM aminocarb (1.20 ± 0.24—fold variation to control, *p* = 0.5497), compared to the control group (1.00 ± 0.18—fold variation to control) (Figure 4—panel B); *NRF1* when TM4 cells were exposed to 0.5 μM aminocarb (1.56 ± 039.—fold variation to control, *p* = 0.2302) or 5 μM aminocarb (1.22 ± 0.34—fold variation to control, *p* = 0.6207), compared to the control group cells (1.00 ± 0.18—fold variation to control) (Figure 4—panel C); *TFAM* when TM4 cells were exposed to 0.5 μM aminocarb (1.14 ± 0.18—fold variation to control, *p* = 0.5571) or 5 μM aminocarb (1.13 ± 0.17—fold variation to control, *p* = 0.5761), compared to the control group cells (1.00 ± 0.13—fold variation to control) (Figure 4—panel D); and mtDNAcn when TM4 cells were exposed to 0.5 μM aminocarb (1.07 ± 0.11—fold variation to control, *p* = 0.6490) or 5 μM aminocarb (1.16 ± 0.13—fold variation to control, *p* = 0.3139), compared to the control group cells (1.00 ± 0.08—fold variation to control) (Figure 4—panel E).

**Figure 5:**
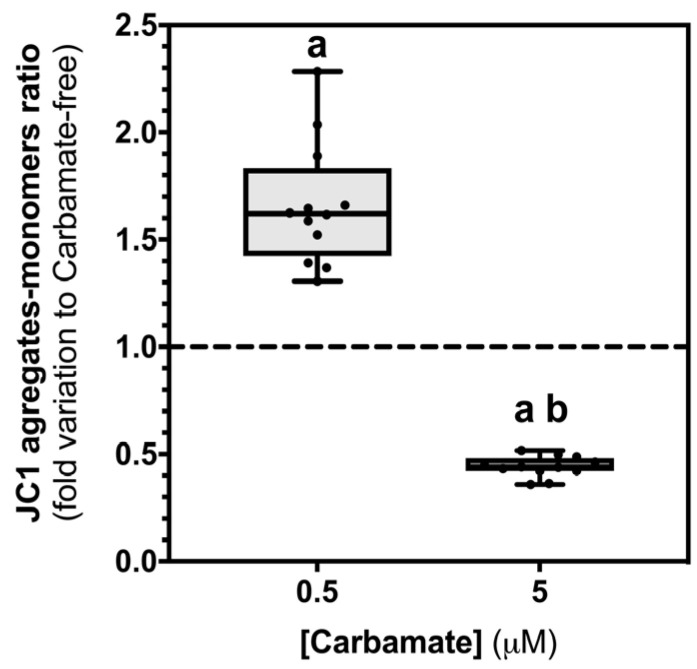
Evaluation of TM4 cells’ ∆ψm in the absence or in the presence of aminocarb (0.5 and 5 μM) following 24 h of treatment, using the dye JC-1. The figure shows pooled data of independent experiments, indicating the JC1 aggregates to monomers ratio of TM4 cells cultured in the absence or in the presence of aminocarb (0.5 and 5 µM). Results were calculated as fold variation to control and are expressed as median ± max/min values (n = 12 for each condition). The dashed line represents the control group. The statistical significance was assessed by a One-way ANOVA followed by Fisher’s LSD test. Significantly different results (*p* < 0.05) are indicated as: a—relative to control group, b—relative to 0.5 µM group.

Despite the fact that we did not observe any significant differences in the expression of the previous mentioned genes, we found it important to better understand the impact of aminocarb on mitochondria. As such, we evaluated ∆ψm, with the JC-1 dye, which is necessary to support mitochondrial biogenesis [32] (Figure 5). Our results demonstrated that the lowest concentration of aminocarb, 0.5 μM (1.66 ± 0.08—fold variation to control, *p* < 0.001), increased the ration J-aggregates/monomers, when compared to the control group cells (1.00 ± 0.02—fold variation to control). On the other hand, exposure to aminocarb 5 μM (0.44 ± 0.01—fold variation to control) decreased this ratio, when compared to both the control group cells (*p* < 0.0001) and the cells exposed to the lowest concentration (*p* < 0.0001).

### 3.5. Exposure to Aminocarb Does Not Induce Alterations in the Protein Levels of Bax and Bcl-2

**Figure 6:**
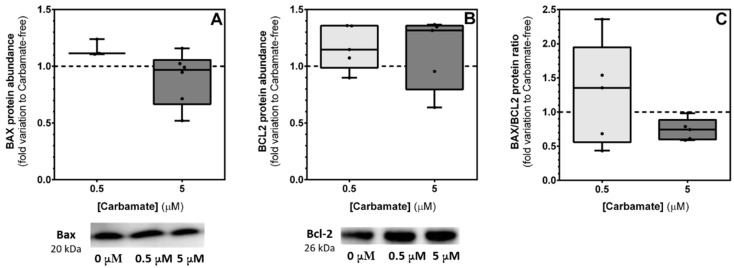
Effect of 0.5 and 5 μM of aminocarb on Bax protein levels (**A**), Bcl-2 protein levels (**B**), and Bax/Bcl-2 protein ratio (**C**) of TM4 cells, following 24 h of treatment. The figure shows pooled data of independent experiments, indicating Bax protein levels, with representative western blot below (**A**), Bcl-2 protein levels, with representative western blot below (**B**), (Supplementary Materials). and Bax/Bcl-2 protein ratio (**C**) of TM4 cells cultured in the absence or in the presence of aminocarb (0.5 and 5 µM). Results were calculated as fold variation to control and are presented as median ± max/min values (n = 6 for each condition). The dashed line represents the control group. The statistical significance was assessed by a RM one-way ANOVA followed by Fisher’s LSD test.

Guided by the results of the cytotoxicity assays, as well as from the JC-1 assay, we hypothesized that exposure to aminocarb may induce TM4 cells to enter the apoptotic process due to mitochondrial dysfunction, via the activation of the mitochondrial pathway [12,13,33]. Thus, we decided to evaluate the levels of the protein Bax, a pro-apoptotic protein, and the protein Bcl-2, an anti-apoptotic protein, both belonging to the mitochondrial pathway [12,13,33]. Our results demonstrated that there were no differences regarding protein levels of Bax or Bcl-2 in TM4 cells exposed to 0.5 μM aminocarb (1.13 ± 0.12 and 1.14 ± 0.08—fold variation to control, *p* = 0.5542 and *p* = 0.2875, respectively) or 5 μM aminocarb (0.89 ± 0.09 and 1.10 ± 0.12—fold variation to control, *p* = 0.5320 and *p* = 0.5407, respectively), when compared to the control group (1.00 ± 0.10 and 1.00 ± 0.05—fold variation to control, respectively) (Figure 6, panels A and B, respectively). Moreover, we calculated the Bax/Bcl-2 protein ratio as an indicator of the apoptotic balance as suggested previously [34], but, once again, no significant alterations in this ratio were observed in TM4 cells exposed to 0.5 μM aminocarb (1.17 ± 0.30—fold variation to control, *p* = 0.6962) or 5 μM aminocarb (0.88 ± 0.15—fold variation to control, *p* = 0.6236), when compared to the control group cells (1.00 ± 0.11—fold variation to control) (Figure 6, panel C).

### 3.6. Exposure to Aminocarb Does Not Alter the Levels of the Protein Caspase-8, but Decreases the Ratio eIF2α-P/eIF2α

**Figure 7:**
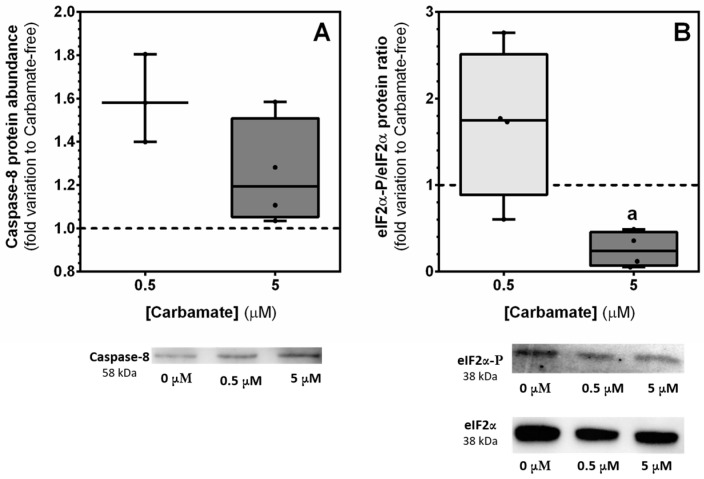
Effect of 0.5 and 5 μM of aminocarb on caspase-8 protein levels (**A**), and eIF2α-P/eIF2α protein ratio (**B**) of TM4 cells, after 24 h of treatment. The figure shows pooled data of independent experiments, indicating caspase-8 protein levels, with representative western blot below (**A**), and eIF2α-P/eIF2α protein ratio, with representative western blot below (**B**) (Supplementary Materials) of TM4 cells cultured in the absence or in the presence of aminocarb (0.5 and 5 µM). Results were calculated as fold variation to control and are presented as median ± max/min values (n = 6 for each condition). The dashed line represents the control group. The statistical significance was assessed by a RM one-way ANOVA followed by Fisher’s LSD test. Significantly different results (*p* < 0.05) are indicated as: a—relative to control.

Since we did not observe significant changes in the levels of proteins associated with the mitochondrial pathway, and it is known that the extrinsic apoptotic pathway is one of the targets of pesticides [35], we evaluated the levels of caspase-8, one of the initiator caspases of the extrinsic apoptotic pathway [13]. However, while our results did not show any significant alterations in the levels of this caspase 8 in cells exposed to 5 µM aminocarb (1.13 ± 0.15—fold variation to control; *p* = 0.6456), we can observe a tendency for increased levels in cells exposed to 0.5 μM aminocarb (1.46 ± 0.10—fold variation to control; *p* = 0.0865), when compared to the control group cells (1.00 ± 0.14—fold variation to control) (Figure 7, panel A).

Taking into consideration the results obtained so far, we decided to evaluate a third possible apoptotic pathway, the ER pathway. In this pathway, the phosphorylation of eIF2α is responsible for the attenuation of ER stress by diminishing protein synthesis [36]. So, we determined the eIF2α-phosphorylation/eIF2α protein ratio and observed that it diminished significantly in cells exposed to 5 μM aminocarb (0.25 ± 0.10—fold variation to control, *p* = 0.0260) when compared to the control group cells (0.95 ± 0.10—fold variation to control) (Figure 7, panel B).

### 3.7. Exposure to 5 µM of Aminocarb Increases the Levels of the Protein Caspase-3 and Promotes Its Activation

**Figure 8:**
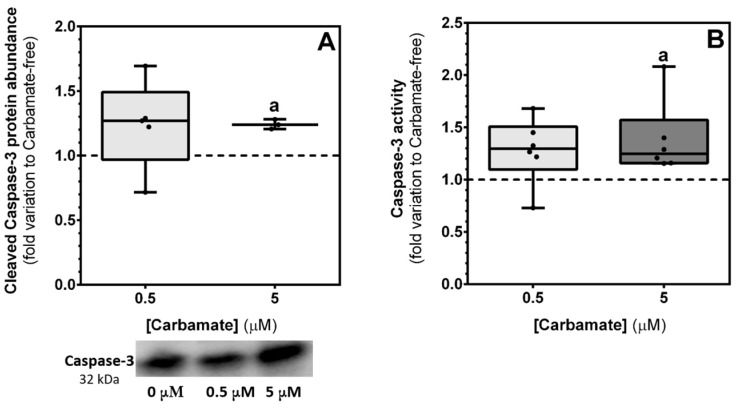
Effect of 0.5 and 5 μM of aminocarb on caspase-3 protein levels (**A**) and on caspase-3 activity (**B**) of TM4 cells, following 24 h of treatment. The figure shows pooled data of independent experiments, indicating caspase-3 protein levels, with representative western blot below (**A**) (Supplementary Materials) and caspase-3 activity (**B**) of TM4 cells cultured in the absence or in the presence of aminocarb (0.5 and 5 µM). Results were calculated as fold variation to control and are presented as median ± max/min values (n = 6 for each condition). The dashed line represents the control group. The statistical significance was assessed by Mixed effect analysis and one-way ANOVA followed by Fisher’s LSD test. Significantly different results (*p* < 0.05) are indicated as: a—relative to control.

In order to corroborate the previous mentioned results, we decided to both evaluate caspase-3 levels and activity after aminocarb treatment, since this executioner caspase represents an irreversible point of the apoptotic cascade [34]. Our results demonstrated that exposure to 5 µM of aminocarb significantly increases the levels of the protein caspase-3 (1.21 ± 0.02—fold variation to control, *p* = 0.0059), when compared to the control group cells (1.00 ± 0.08—fold variation to control) (Figure 8, panel A). Similarly, exposure to the same aminocarb concentration demonstrated a significant increase in caspase-3 activity (1.38 ± 0.14—fold variation to control, *p* = 0.0487), when compared to the control group (1.00 ± 0.10—fold variation to control) (Figure 8, panel B).

### 3.8. Exposure to Aminocarb Does Not Induce Oxidative Stress

**Figure 9:**
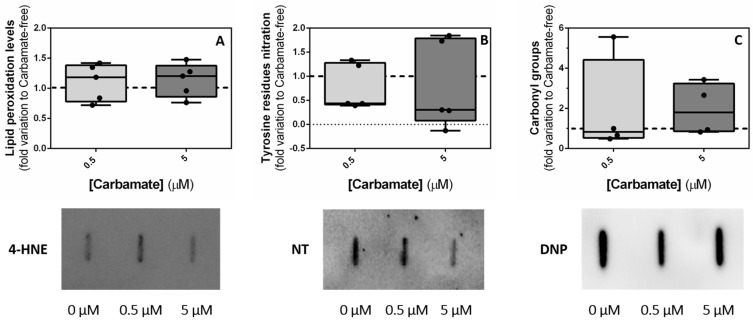
Oxidative stress-induced damage in TM4 cells in the absence or in the presence of aminocarb (0.5 and 50 μM) on lipid peroxidation, with representative slot-blot below (**A**), tyrosine residues nitration, with representative slo-blot below (**B**), and carbonyl group formation, with representative slo-blot below (**C**), after 24 h of treatment. Results were calculated as fold variation to control and are expressed as median ± max/min values (n = 4–6 for each condition). The dashed line represents the control group. The statistical significance was assessed by a One-way ANOVA followed by Fisher’s LSD test.

Pesticides are known to induce the production of reactive oxygen species (ROS), which lower the antioxidant levels and their defense against oxidative damage. In this way, lipids, proteins and nucleic acids are targeted, due to the imbalance, and cellular signalling pathways are affected [37]. Thus, we decided to evaluate possible lipid and protein oxidative modifications caused by exposure to aminocarb. Nevertheless, our results did not show any significant alterations in lipid peroxidation when TM4 cells were exposed to 0.5 μM aminocarb (1.10 ± 0.14—fold variation to control, *p* = 0.6169) or 5 μM aminocarb (1.14 ± 0.13—fold variation to control, *p* = 0.5071) as compared with the control group (1.00 ± 0.16—fold variation to control) (Figure 9—panel A), protein nitration when TM4 cells were exposed to 0.5 μM aminocarb (0.76 ± 0.21—fold variation to control, *p* = 0.5722) or 5 μM aminocarb (0.81 ± 0.41—fold variation to control, *p* = 0.6449) as compared with the control group cells (1.00 ± 0.19—fold variation to control) (Figure 9—panel B), or protein oxidation, determined by protein carbonyl groups content, when TM4 cells were exposed to 0.5 μM aminocarb (1.93 ± 1.22—fold variation to control, *p* = 0.4316) or 5 μM aminocarb (1.96 ± 0.64—fold variation to control, *p* = 0.4149) as compared with the control group cells (1.00 ± 0.11—fold variation to control) (Figure 9—panel C).

## 4. Discussion

Aminocarb is an insecticide that belongs to the carbamate family, one of the most widely used classes of pesticides worldwide [16,38]. Notwithstanding, studies on the impact of this compound on male fertility are scarce. As such, and due to the crucial roles of sustentacular Sertoli cells in the maintenance of spermatogenesis and, consequently, in the normal function of the male reproductive system, we decided to study the impact of aminocarb on these cells, specifically on a mouse Sertoli cell line, TM4. We started by assessing the cytotoxicity caused by exposure to increasing concentrations of aminocarb. From our experiments we could conclude that exposure to the highest concentrations of aminocarb (5, 50 and 500 μM) exerts an antiproliferative effect, a consequence that may be particularly impactful for male fertility, since the number of sustentacular Sertoli cells determines the number of germ cells supported [39]. Moreover, we also observed a reduction in the metabolic viability of TM4 cells exposed to most of the doses assayed, thus corroborating the previous results, and highlighting the deleterious effects of aminocarb on sustentacular Sertoli cells. Consequently, this suggests an association of this compound with compromised male reproductive potential. Previous studies that evaluated the effect of other pesticides on male fertility reported similar results. For instance, a study in which the TM4 cell line was also used, cells were exposed to 10, 20, 40 and 80 μM of cypermethrin, a synthetic pyrethroid pesticide, and the results obtained showed that this compound can diminish cells proliferation and viability [40]. In a different study, with the organochlorine pesticide technical-grade chlordane, the authors demonstrated the ability of pesticides to decrease the proliferation and viability of cells from a rat Sertoli cell line, SER-W3. However, contrary to our study, increased cytolysis was also reported for the highest concentrations (10 and 1000 nM) [41].

The reduced proliferation of sustentacular Sertoli cells and, particularly, the reduced metabolic viability observed in this study suggested a possible mitochondrial dysfunction. In this way, in order to better understand the impact of aminocarb on TM4 cell mitochondria, we quantified the mRNA transcripts of genes involved in mitochondrial biogenesis like *SIRT1*, *PGC-1α*, *NRF1* and *TFAM*. These form a pathway that starts with the deacetylation of PGC-1α by the NAD^+^ -dependent protein deacetylase sirtuin 1 (SIRT1) [42], which in turn can coactivate and enhance the expression and activity of several transcription factors, including the nuclear respiratory factor 1, NRF1. This regulates the activation of the mitochondrial transcription factor A (TFAM) [43], which is responsible for the expression of mtDNA transcription [44]. Yet, our results did not show any significant difference in any of the transcripts studied. Moreover, we quantified the mtDNA content, but, once again, no significant differences were found between the experimental groups. Despite the fact we did not observe significant changes in the mRNA levels of genes associated with mitochondrial biogenesis, we found it important to assess ∆ψm, after aminocarb treatment, since this parameter is necessary to support mitochondrial function [32]. In this way, we measured the JC-1 aggregates to monomers ratio and found that the two concentrations tested presented opposing results. Cells exposed to the 0.5 μM concentration showed an increased JC-1 ratio, indicating polarized mitochondria, while those exposed to the concentration of 5 μM showed a decreased JC-1 ratio, suggesting unpolarized unhealthy mitochondria [45]. This reduction in the polarized mitochondria of TM4 cells, together with the reduced proliferation and decreased metabolic viability led us to hypothesize a promotion of apoptosis by exposure to the highest concentration (5 μM) of aminocarb. Hence, we evaluated the expression or activity of selected markers of the distinct apoptotic pathways and of final apoptosis effectors.

It is known that mitochondria exert a crucial role in releasing several important apoptosis, inducing molecules as a result of permeabilization of mitochondrial membrane. Both permeabilization and integrity of mitochondrial membrane are maintained by members of the B cell lymphoma (Bcl)-2 family, which can act either as pro-apoptotic (Bax, Bak, Bid, Bim, Puma, Noxa, Bad and Blk) or anti-apoptotic (Bcl-2, Bcl-XL, Bcl-X and BAG), and it is this balance between pro- and anti-apoptotic molecules that keeps cellular homeostasis and determines cells’ fates [13]. In this way, we decided to evaluate the levels of the pro-apoptotic protein, Bax, and the anti-apoptotic protein, Bcl-2, after exposure to aminocarb, but no significant changes were observed in the levels of either of these two proteins. As the balance between Bax and Bcl-2 expression is crucial in determining the fate of a cell, dictating whether it will survive or undergo apoptosis in response to stimuli, we also calculated the Bax/Bcl-2 protein ratio. A higher Bax/Bcl-2 ratio reduces cellular resistance to apoptotic signals through the intrinsic pathway, thereby promoting increased cell death, while a lower ratio of Bax to Bcl-2 suggests higher cellular resilience against apoptotic triggers, diminishing the likelihood of cell death [46]. Our results also showed no significant changes in this ratio, which could indicate a balance between pro- and anti-apoptotic molecules, and argue against the participation of this mitochondrial dependent apoptotic pathway.

Bearing in mind the results of the cytotoxicity assays and JC-1 mentioned above, we decided to explore other apoptotic pathways that may be activated by aminocarb, and explored the extrinsic apoptotic pathway, which is one of the targets of pesticides, as mentioned before [35]. We evaluated the expression of caspase-8, which mediates the cleavage and activation of effector caspases [14]. Notwithstanding, our results did not show any significant differences in the expression of this marker, which made us conclude that the possible initiation of apoptosis in our study may be due to other pathways. In fact, exposure to adverse environmental stimuli, like environmental toxins, can disturb ER homeostasis, leading to the accumulation of misfolded or unfolded proteins in the ER lumen, and consequently creating a phenomenon known as ER stress [47]. In this situation, a survival response known as the Unfolded Protein Response (UPR) is activated that either promotes cell survival or death, depending on the severity of the ER stress. The UPR involves three signaling mechanisms, one of them being the transient inhibition of translation. This mechanism is controlled by protein kinase RNA (PKR)-like ER kinase (PERK) that once activated phosphorylates the eukaryotic initiation factor 2α (eIF2α) that is involved in general inhibition of the initiation of mRNA translation. On the other hand, phosphorylated eIF2α specifically upregulates a cascade of events that leads to the translation of genes involved in amino acid biosynthesis and transport, as well the antioxidant response. However, under conditions of severe ER stress, ATF-4 activates a pathway that leads to the dephosphorylation of eIF2α [48]. In our study, we evaluated the protein ratio eIF2α-P/eIF2α, and observed a decrease in cells exposed to 5 μM of aminocarb, which means that there is an imbalance between the levels of the protein eIF2α and its phosphorylated counterpart, promoting the expression of eIF2α, and so ER stress. As such, in order to corroborate these results, we decided to evaluate both the levels and activity of caspase-3, since the activation of this effector caspase represents an irreversible point in the apoptotic process [34]. In fact, caspase-3 is a cysteine-aspartic acid protease which belongs to the group of executioner caspases, that once activated promotes the cleavage of key structural proteins, cell cycle proteins and DNase proteins, resulting in the blebbing and condensing of cells leading to cell death [49]. Our results showed that, indeed, 5 μM of aminocarb increased both the levels of cleaved caspase-3 protein (activated form), as of caspase-3 activity, leading us to conclude, that 5 μM of aminocarb induces cell death by ER stress. In line with our findings, studies with other environment contaminants on sustentacular Sertoli cells have also demonstrated the capacity of those chemicals to induce ER stress and apoptosis. In fact, a recent study from Shen and colleagues showed that exposure to increasing concentrations (0, 10, 20, 40 and 80 μM) of cypermethrin, a synthetic pyrethroid used as an insecticide, caused apoptosis of TM4 cells due to ER stress through the eIF2α-ATF4 pathway [50]. Similarly, a previous study reported that exposure of rat sustentacular Sertoli cells to graded concentrations (0, 0.1, 1, 10, 20 and 30 μM) of nonylphenol, a nonionic surfactant that is widespread release into aquatic environments, lead these cells to apoptosis, also due to ER stress [51]. Similarly, various studies with other cells exposed to pesticides reported ER stress and apoptosis, as for example in the study from Goswami and colleagues, in which a correlation between rotenone pesticide and ER stress that conducted neuro-2A cells to death was observed [52]. Finally, since exposure to xenobiotics and other environmental factors can lead to an overproduction of ROS in intra- or extracellular spaces, thus causing damage to biomolecules and leading to cellular dysfunction and apoptosis [15], we decided to evaluate possible lipid and protein modifications caused by exposure to aminocarb. Thus, we assessed levels of lipid peroxidation, protein nitration and protein carbonylation, but none of the results showed significant differences among the experimental groups.

## 5. Conclusions

In conclusion, this study demonstrated that exposing TM4 cells to aminocarb resulted in significant cytotoxic effects, with the 5 μM concentration inducing depolarization of ∆ψm, and specifically triggering ER stress, leading to cells’ apoptosis through the disruption of the ER stress pathway. These findings make a significant contribution to understanding the impact of the pesticide aminocarb on sustentacular Sertoli cells function and homeostasis. Moreover, they shed light on potential causes of male infertility associated with exposure to xenobiotics, revealing a novel mechanism of action for aminocarb that could compromise testicular cell physiology and, consequently, male fertility. Current studies are in progress to better understand the impact of aminocarb on other testicular cells, and therefore on male fertility overall.

## Figures and Tables

**Table 1:** List of targets and respective primers used in this study.

Gene	Primer Sequence (5′-3′)	AT (°C)	Efficency (%)
Sirt1	Sense: ACAGAACGTCACACGCCAG Anti-Sense: ACAATCTGCCACAGCGTCATA	58	80
PGC-1α	Sence: TGTGTGTCAGAGTGGATTGGAG Anti-Sence: GCAGGCTCATTGTTGTACTGG	56	82.4
NRF1	Sence: GCTGCAGGTCCTGTGGGAAT Anti-Sence: ACTCAAACACATGAGGCCGT	64	98.7
TFAM	Sence: GATGGGTATGGAGAAGGAGGC Anti-Sence: CCCTGAGCCGAATCATCCTTT	56	88.9
β2M	Sence: ACGTAACACAGTTCCACCCG Anti-Sence: TCTCGATCCCAGTAGACGGT	58	110
ND1	Sence: GCATCTTATCCACGCTTCCG Anti-Sence: TGGTGGTACTCCCGCTGTAA	58	N.A.
β2M_nc_	Sence: GCTCACACTGAATTCACCCC Anti-Sence: CGGCCATACTGGCATGCTTA	58	N.A.

Legend: Sirt1—Sirtuin 1; PGC-1α—peroxisome proliferator-activated receptor γ coactivator 1-α; NRF1—nuclear respiratory factor 1; TFAM—transcription factor A, mitochondrial; β2M—β2-Microglobulin; ND1—NADH dehydrogenase subunit 1; β2M_nc_—nuclear encoded β2-microglobulin; AT—annealing temperature; N.A.—non applicable.

**Table 2:** List of the primary and secondary antibodies used in this study.

Antibody	Host Specie	Molecular Weight (kDa)	Dilution	Vendor	Catalog
Bax	Rabbit	21	1:1000	Abcam, United Kingdom	ab32503
Bax	Rabbit	20	1:1000	Cell Signaling Technology, MA, USA	2772S
Bcl-2	Rabbit	26	1:1000	Abcam, United Kingdom	ab196495
Cleaved Caspase-3	Rabbit	17	1:1000	Sigma-Aldrich, Germany	pc679
Caspase-8	Rabbit	58	1:500	Abcam, United Kingdom	ab138485
eIF2α	Rabbit	38	1:1000	Cell Signaling, MA, USA	97225
Phospho-eIF2α (Ser51)	Rabbit	38	1:1000	Cell Signaling, MA, USA	119A11
Rabbit	Goat	-	1:1000	Sigma-Aldrich, Germany	A3687

## Data Availability

The original contributions presented in the study are included in the article/Supplementary Materials, further inquiries can be directed to the corresponding author/s.

## Supplementary Materials

The following supporting information can be downloaded at: https://www.mdpi.com/article/10.3390/biology13090721/s1, WB figures.
