# Supplementary material for: Aminocarb Exposure Induces Cytotoxicity and Endoplasmic Reticulum Stress-Mediated Apoptosis in Mouse Sustentacular Sertoli Cells: Implications for Male Infertility and Environmental Health
Source: Biology (Basel). 2024 Sep 14;13(9):721. doi: 10.3390/biology13090721 (PMC11429014; doi:10.3390/biology13090721)
Supplement: Supplementary file 1 [file biology-13-00721-s001.zip › biology-3149276-supplementary.pdf]

Legend

**Protein marker**	M
**Control (0 μM of aminocarb)**	C
**0.5 μM of aminocarb**	[0.5]
**5 μM of aminocarb**	[5]

- Limited with the blue box are the bands considered!

Bax protein

N1

M Total protein $M – C_1$ - $[0.5]_1$ - $[5]_1$

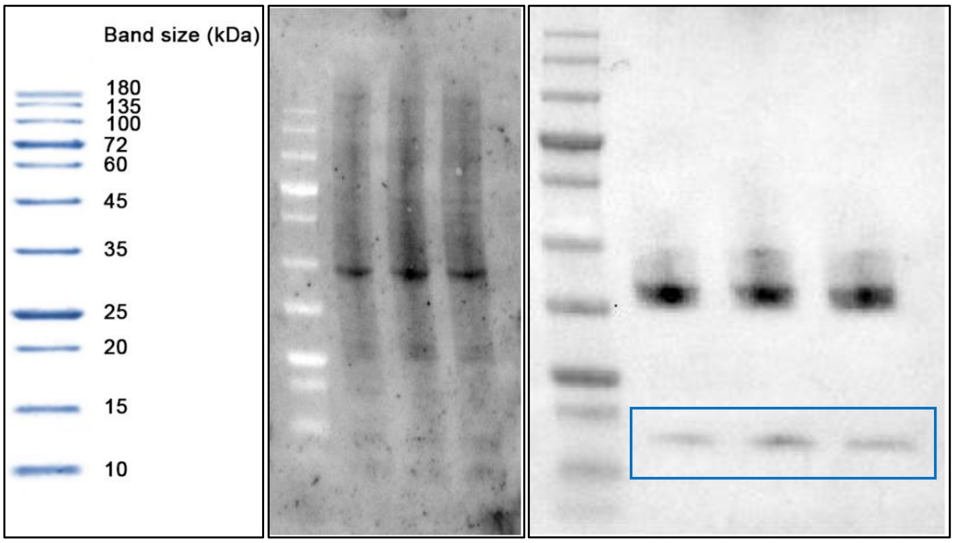

≈ 20 KDa

N2 & N3

M Total protein

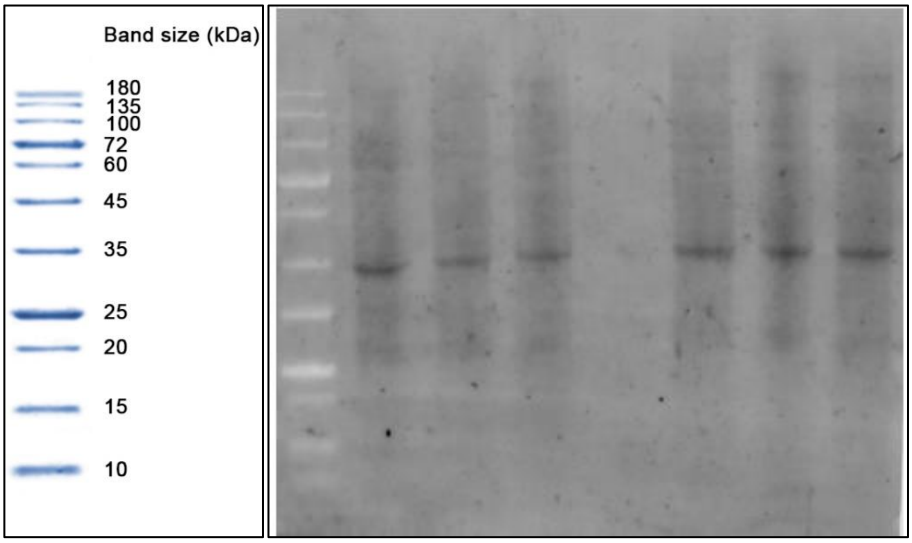

M – C_2 - $[0.5]_2$ - $[5]_2$ – C_3 - $[0.5]_3$ - $[5]_3$

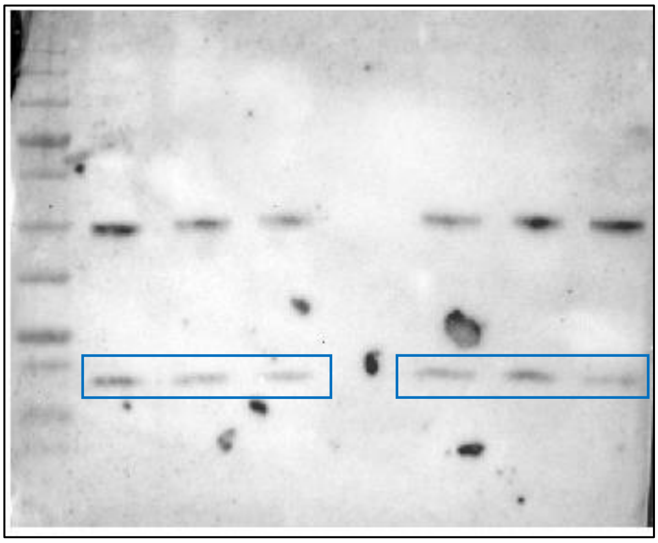

≈ 20 KDa

N4, N5 & N6

M Total protein

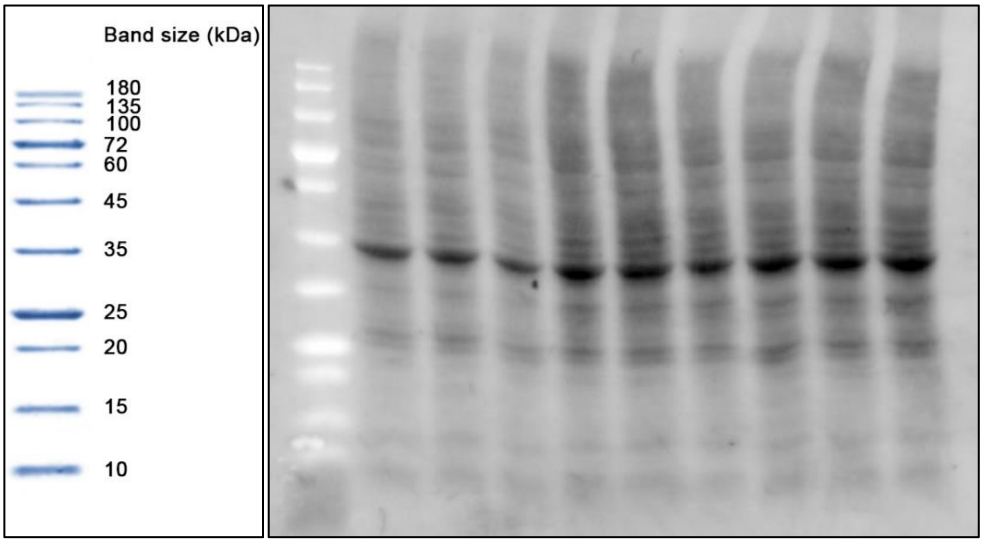

M – C_4 - $[0.5]_4$ - $[5]_4$ – C_5 - $[0.5]_5$ - $[5]_5$ – C_6 - $[0.5]_6$ - $[5]_6$

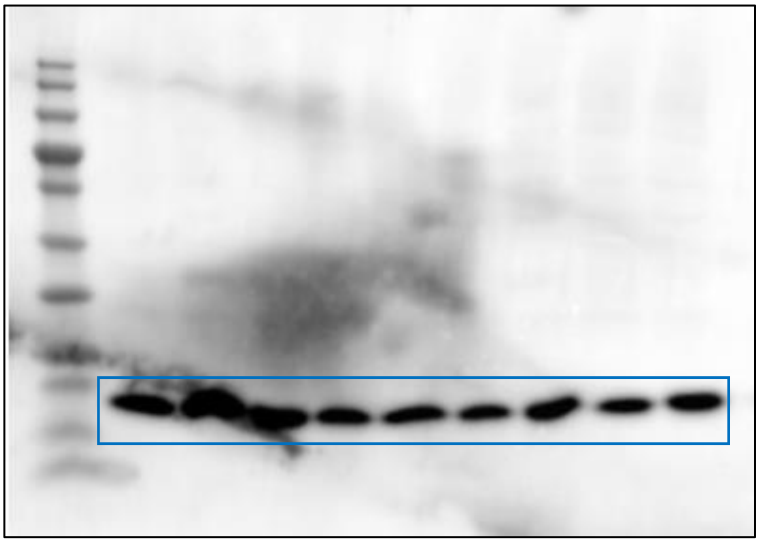

≈ 20 KDa

Bcl-2 protein

N1, N2 & N3

M Total protein

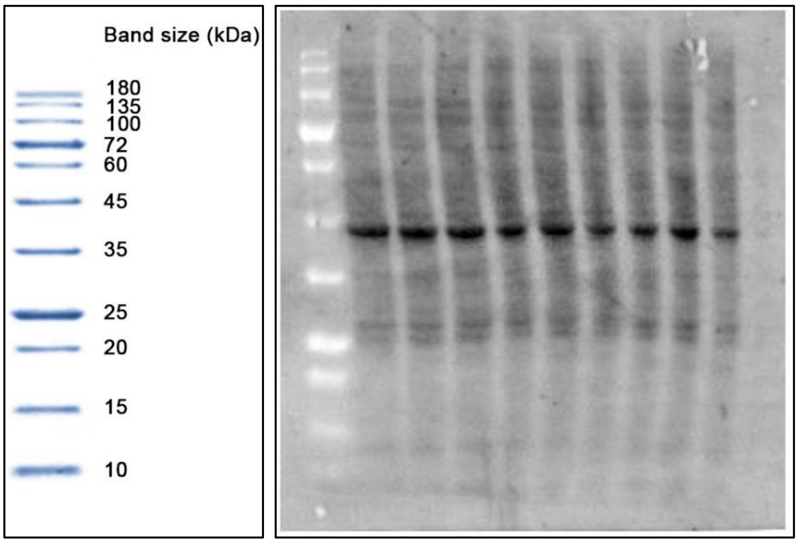

M–C_1-$[0.5]_1$-$[5]_1$–C_2-$[0.5]_2$-$[5]_2$–C_3-$[0.5]_3$-$[5]_3$

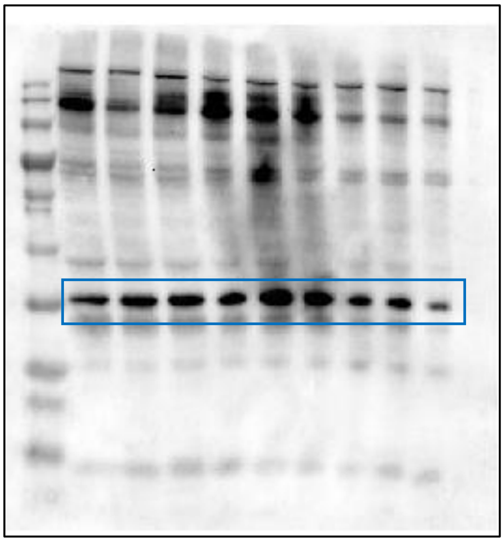

≈ 26 KDa

N4

M Total protein

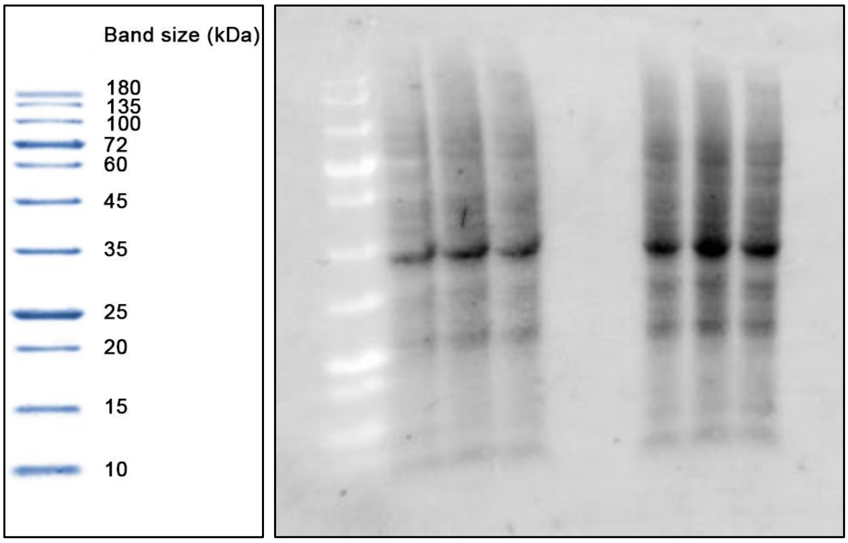

M–NC–NC–NC–C_4-$[0.5]_4$-$[5]_4$ **(NC - band Not Considered)**

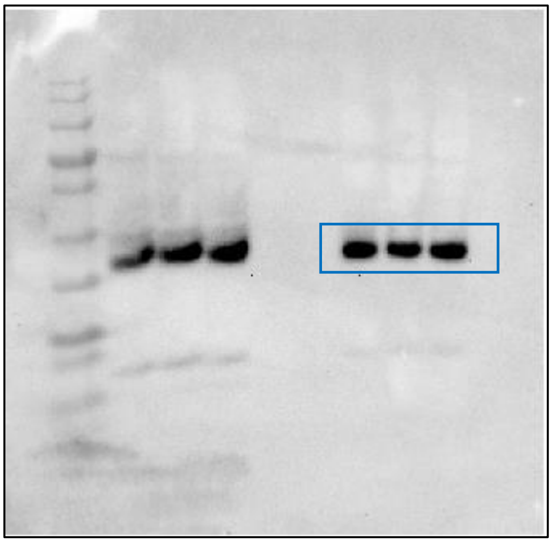

≈ 26 KDa

N5 & N6

M Total protein

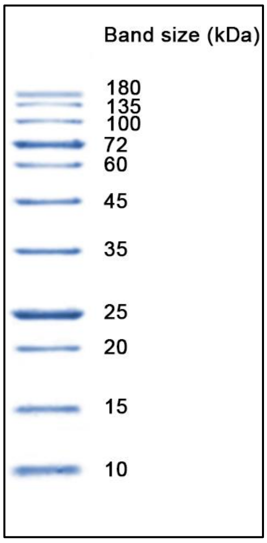

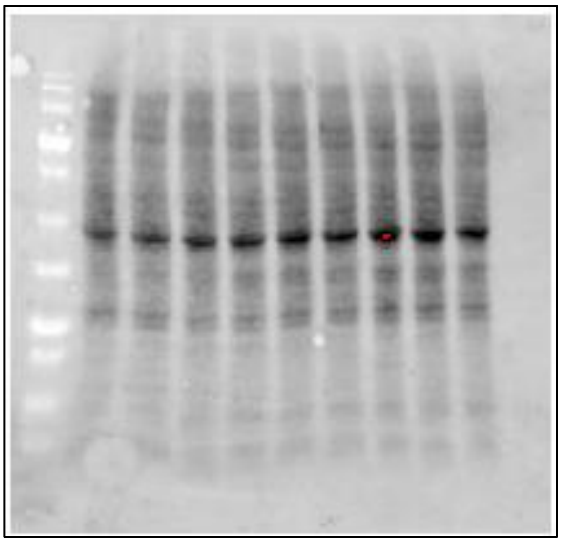

M–NC–NC–NC–C_5-$[0.5]_5$-$[5]_5$–C_6-$[0.5]_6$-$[5]_6$

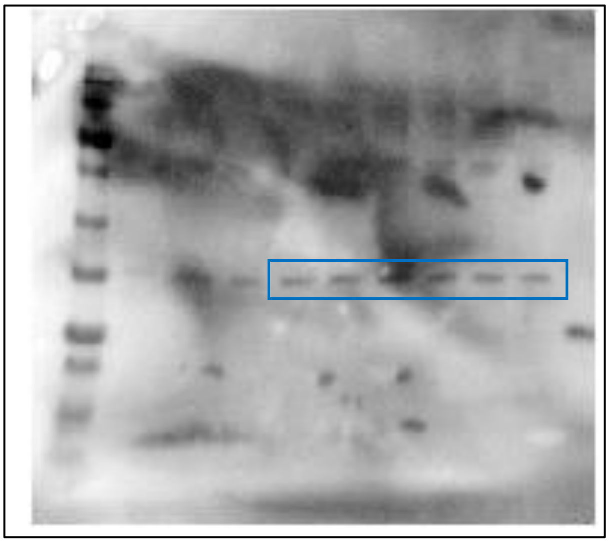

≈ 26 KDa

Caspase-3 protein

N1

M — Total protein — M – C_1 - $[0.5]_1$ - $[5]_1$

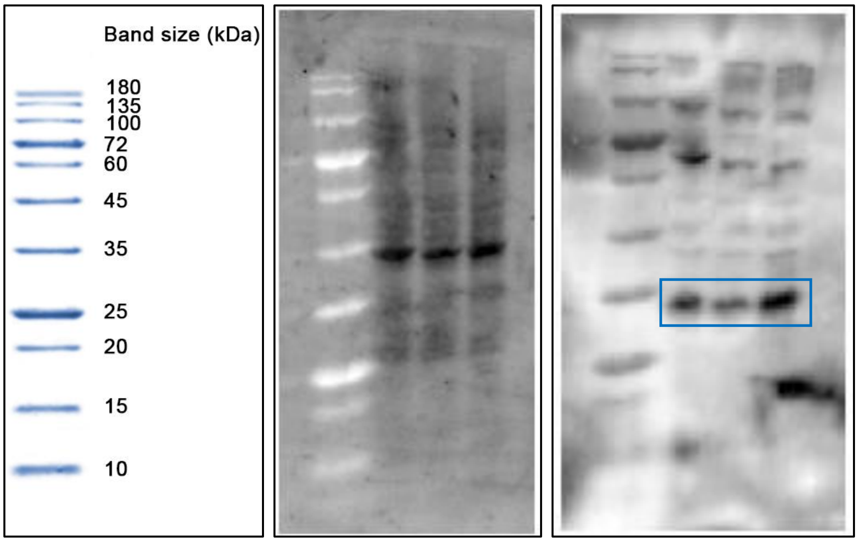

≈ 32 KDa

N2 & N3

M — Total protein — M–C_2-$[0.5]_2$-$[5]_2$–C_3-$[0.5]_3$-$[5]_3$

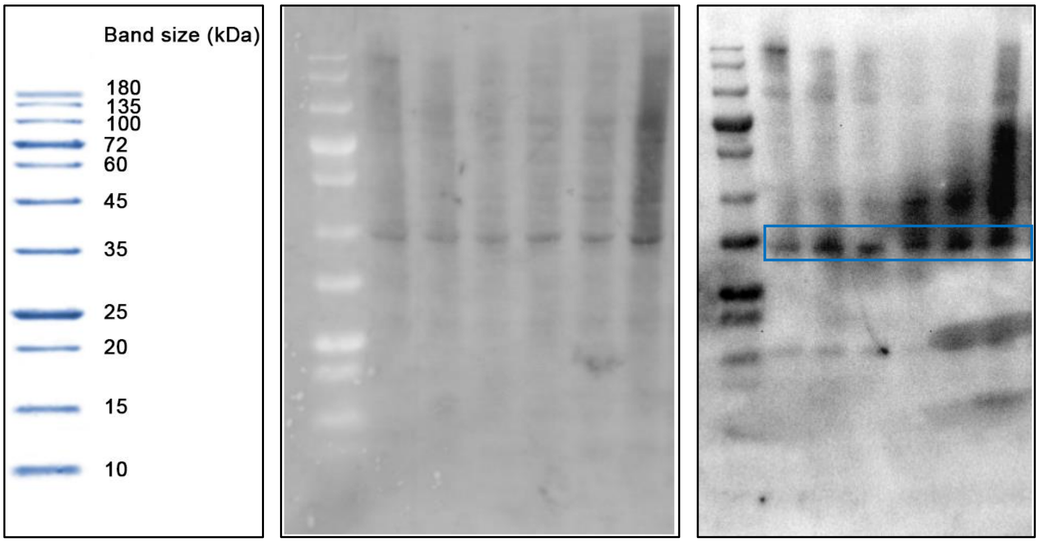

≈ 32 KDa

N4, N5 & N6

M Total protein

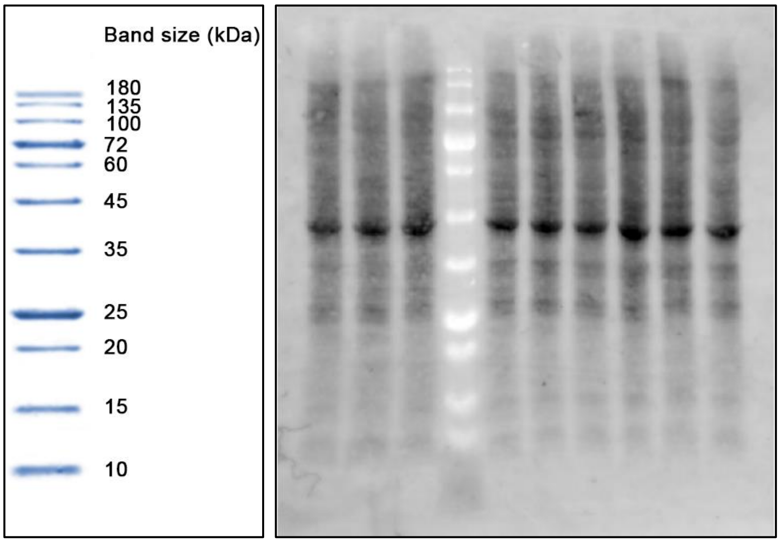

C_4-$[0.5]_4$-$[5]_4$-M–C_5-$[0.5]_5$-$[5]_5$-$[5]_6$-$[0.5]_6$-C_6

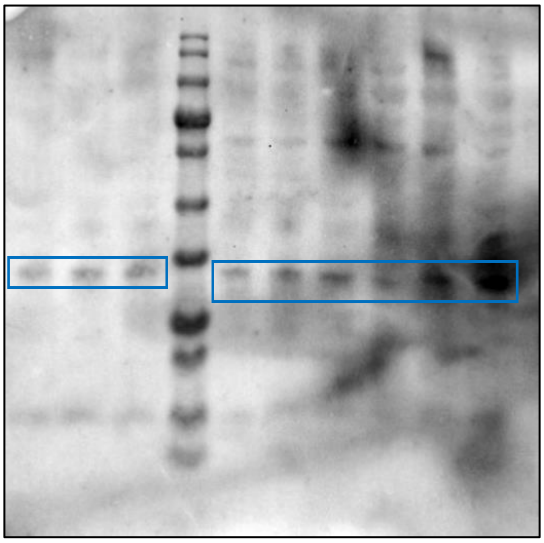

≈ 32 KDa

Caspase-8 protein

N1 & N2

M Total protein

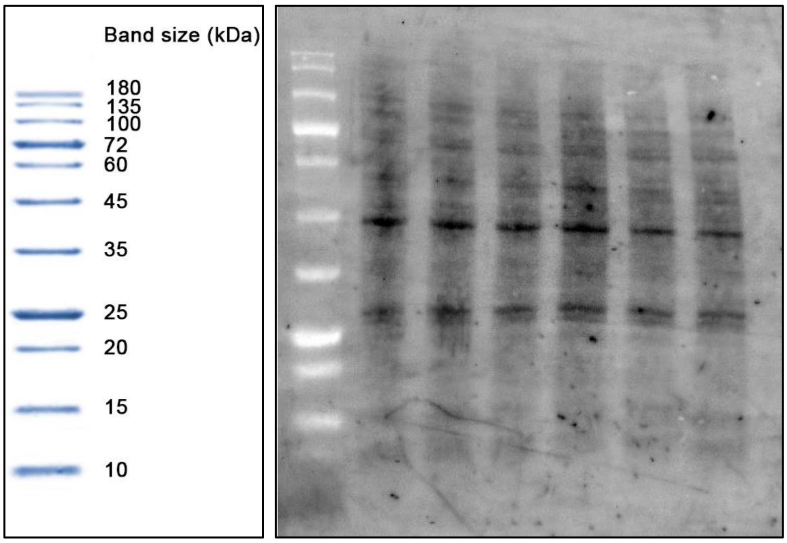

$M–C_1-[0.5]_1-[5]_1-C_2-[0.5]_2-[5]_2$

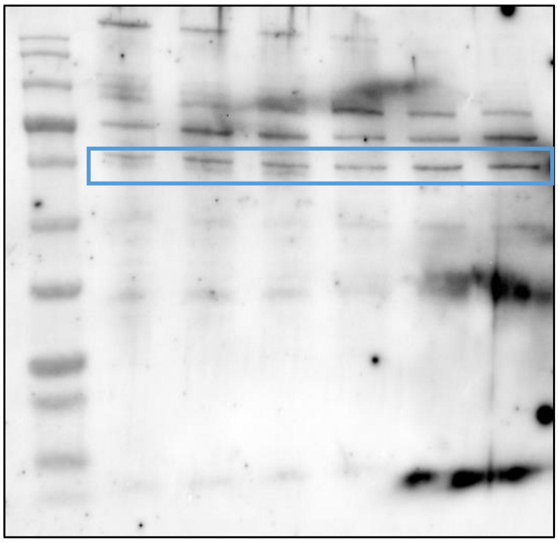

≈ 58 KDa

N3

M Total protein

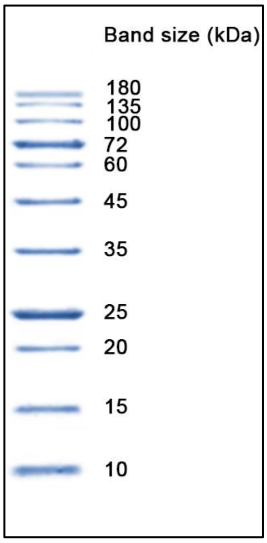

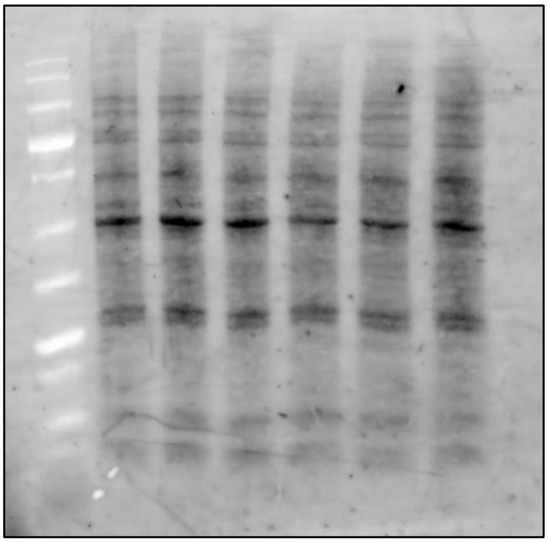

M–NC-NC-NC-C_3-$[0.5]_3$-$[5]_3$

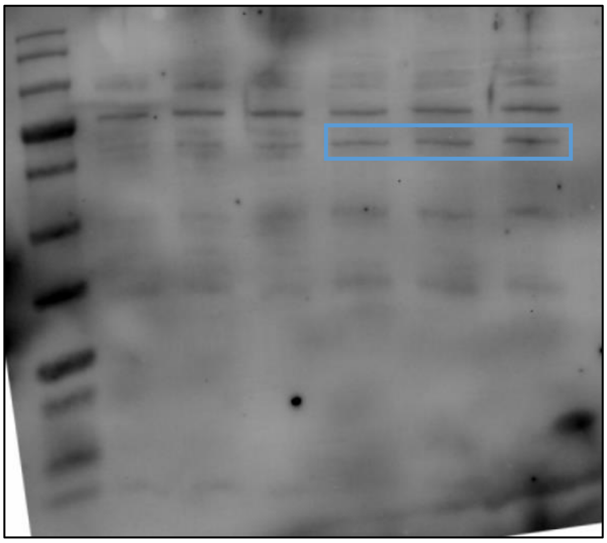

≈ 58 KDa

N4

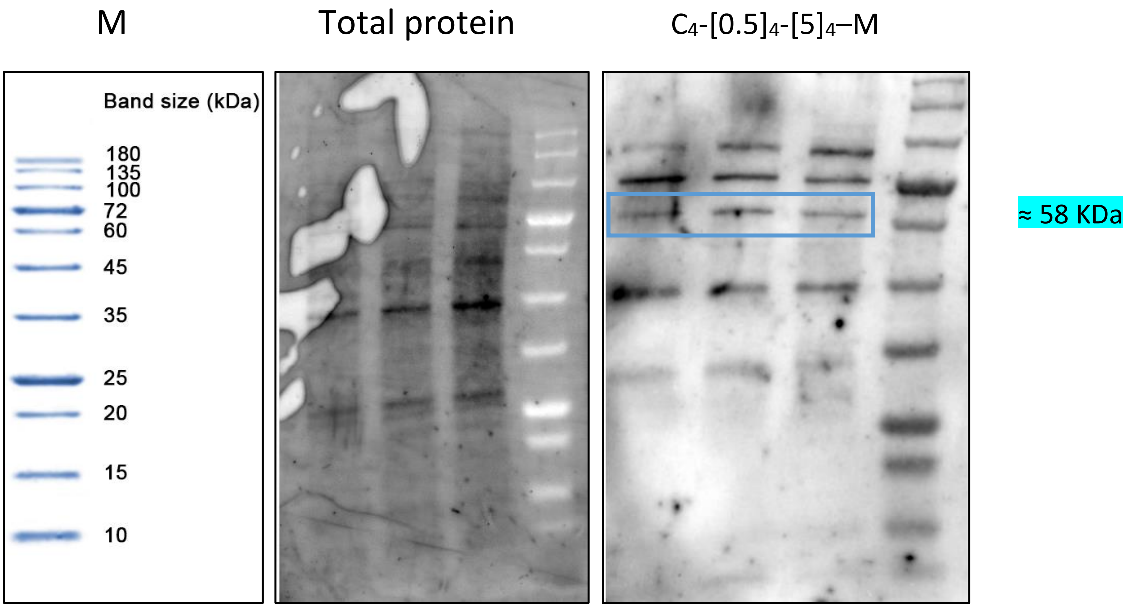
M
Total protein
C4-[0.5]4-[5]4–M
Band size (kDa)
180
135
100
72
60
45
35
25
20
15
10
≈ 58 KDa

N5 & N6

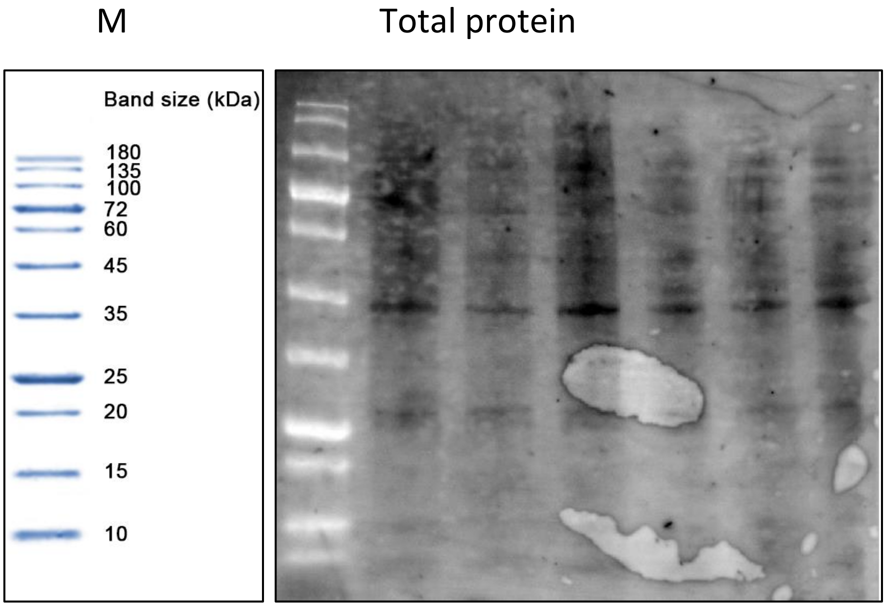
M
Total protein
Band size (kDa)
180
135
100
72
60
45
35
25
20
15
10

M–C_5-$[0.5]_5$-$[5]_5$–C_6-$[0.5]_6$-$[5]_6$

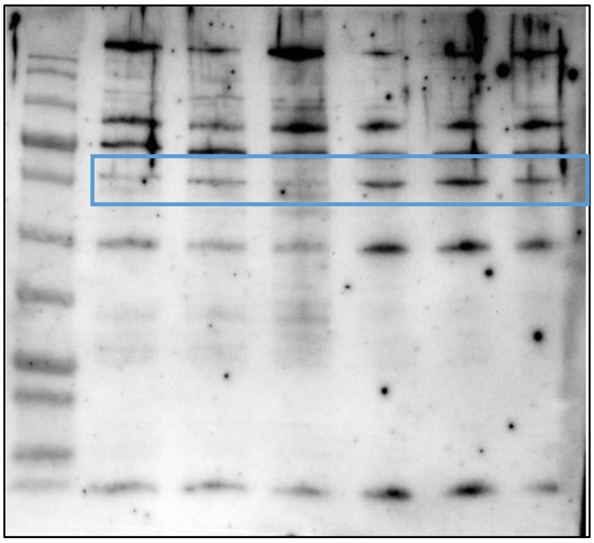

≈ 58 KDa

eIF2α protein

N1

M

Total protein

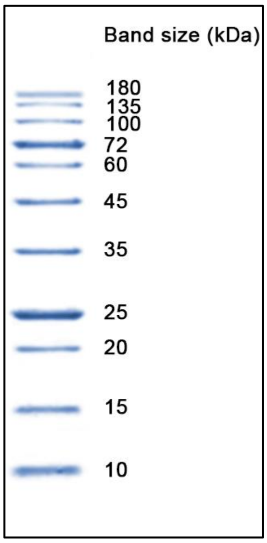

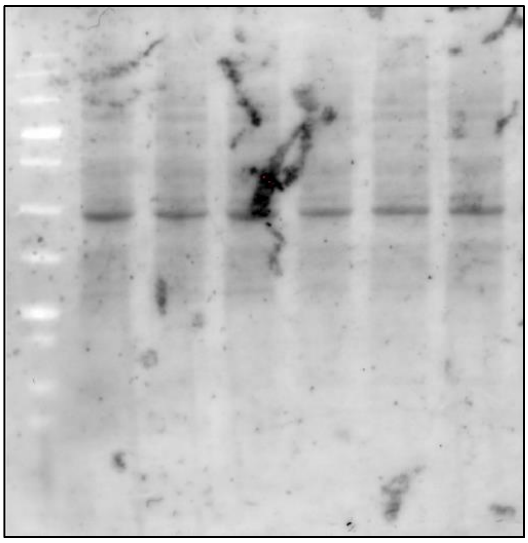

M–NC-NC-NC–C_1-$[0.5]_1$-$[5]_1$

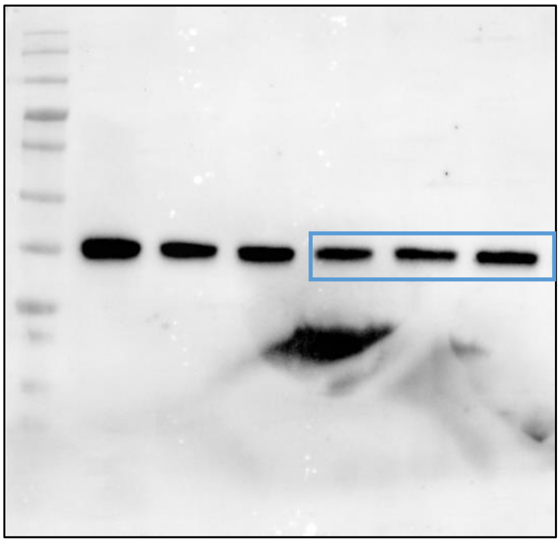

≈ 38 KDa

N2 & N3

M   Total protein

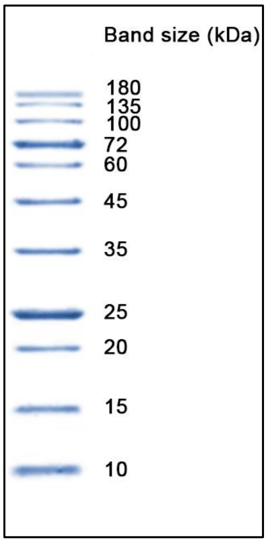

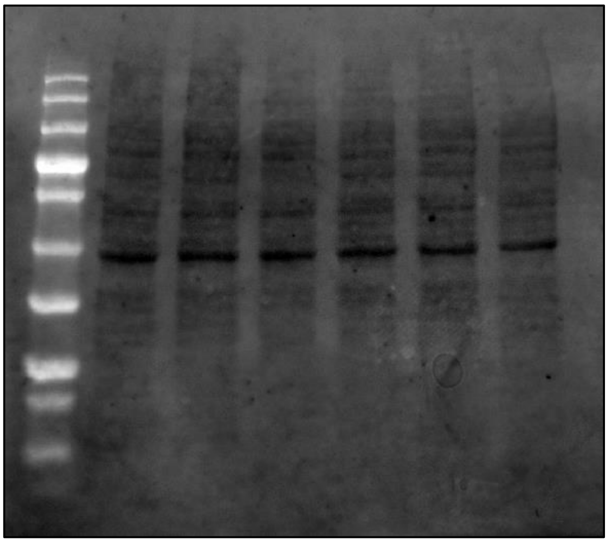

$M–C_2$-$[0.5]_2$-$[5]_2$–C_3-$[0.5]_3$-$[5]_3$

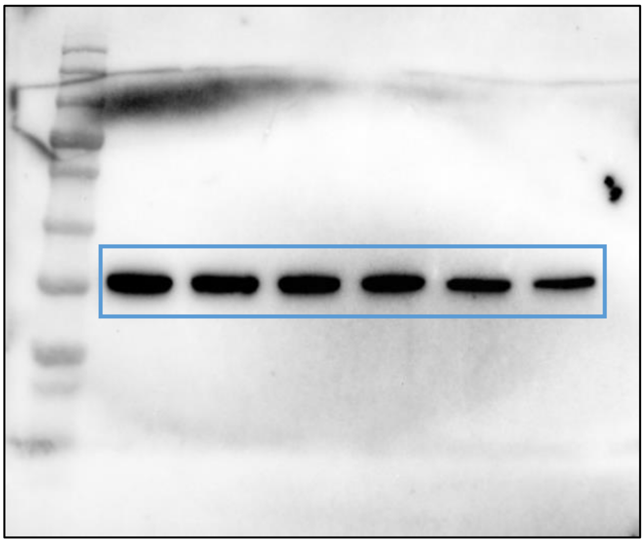

≈ 38 KDa

eIF2α phosphorylation protein

<u>N1 & N2</u>

M Total protein

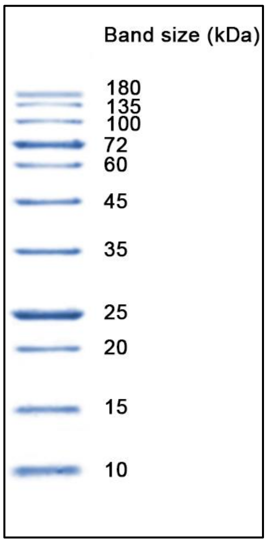

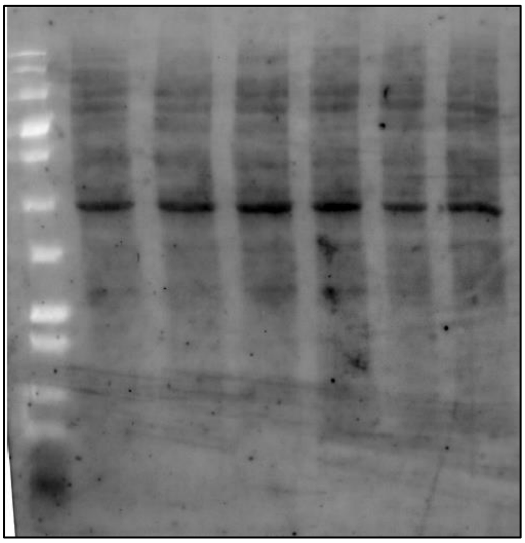

M–C_1-$[0.5]_1$-$[5]_1$–C_2-$[0.5]_2$-$[5]_2$

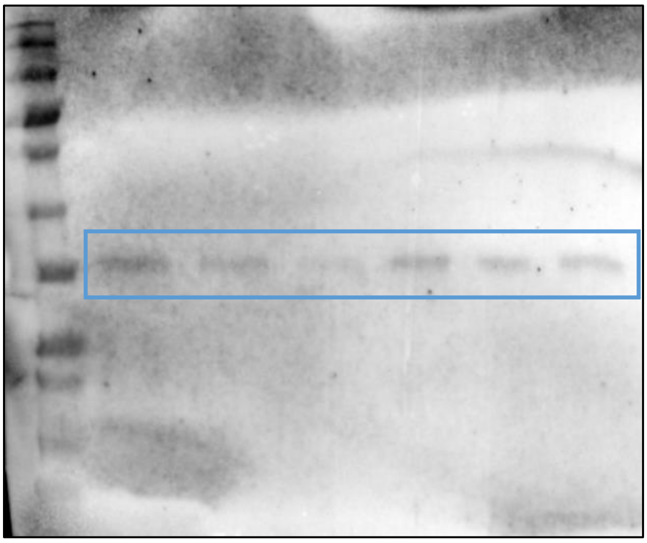

≈ 38 KDa

N3

M Total protein

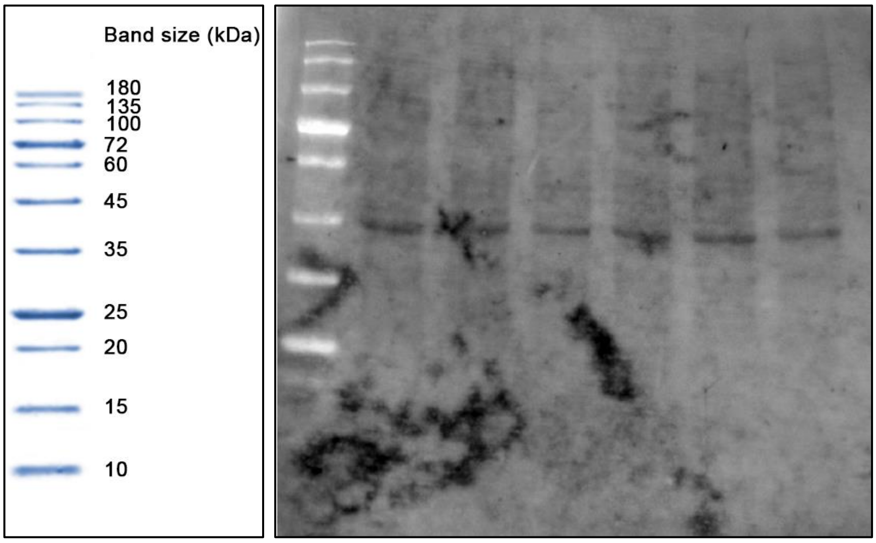

M–NC-NC-NC–C_3-$[0.5]_3$-$[5]_3$

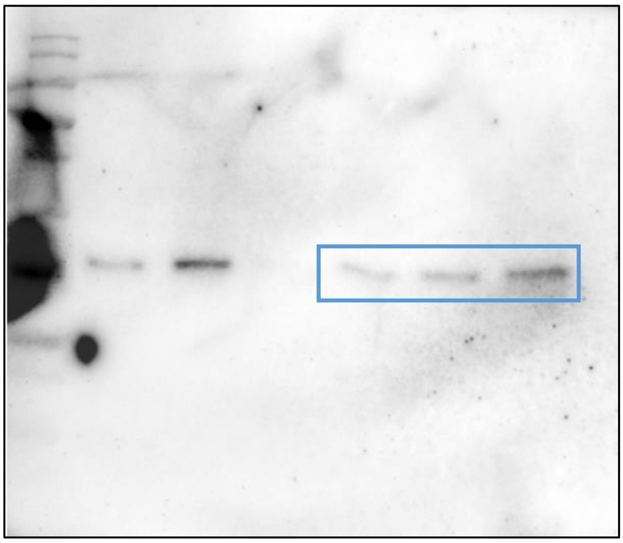

≈ 38 KDa
